# Underlying chronic inflammation alters the profile and mechanisms of acute neutrophil recruitment

**DOI:** 10.1002/path.4776

**Published:** 2016-10-19

**Authors:** Bin Ma, James R Whiteford, Sussan Nourshargh, Abigail Woodfin

**Affiliations:** ^1^ William Harvey Research Institute, Barts and the London School of Medicine and DentistryQueen Mary University of LondonLondonUK; ^2^ Cardiovascular DivisionKing's College LondonLondonUK

**Keywords:** acute inflammation, chronic inflammation, ischaemia, neutrophil, monocyte, macrophage, angiogenesis

## Abstract

Chronically inflamed tissues show altered characteristics that include persistent populations of inflammatory leukocytes and remodelling of the vascular network. As the majority of studies on leukocyte recruitment have been carried out in normal healthy tissues, the impact of underlying chronic inflammation on ongoing leukocyte recruitment is largely unknown. Here, we investigate the profile and mechanisms of acute inflammatory responses in chronically inflamed and angiogenic tissues, and consider the implications for chronic inflammatory disorders. We have developed a novel model of chronic ischaemia of the mouse cremaster muscle that is characterized by a persistent population of monocyte‐derived cells (MDCs), and capillary angiogenesis. These tissues also show elevated acute neutrophil recruitment in response to locally administered inflammatory stimuli. We determined that Gr1^low^ MDCs, which are widely considered to have anti‐inflammatory and reparative functions, amplified acute inflammatory reactions via the generation of additional proinflammatory signals, changing both the profile and magnitude of the tissue response. Similar vascular and inflammatory responses, including activation of MDCs by transient ischaemia–reperfusion, were observed in mouse hindlimbs subjected to chronic ischaemia. This response demonstrates the relevance of the findings to peripheral arterial disease, in which patients experience transient exercise‐induced ischaemia known as claudication.These findings demonstrate that chronically inflamed tissues show an altered profile and altered mechanisms of acute inflammatory responses, and identify tissue‐resident MDCs as potential therapeutic targets. © 2016 The Authors. The Journal of Pathology published by John Wiley & Sons Ltd on behalf of Pathological Society of Great Britain and Ireland.

## Introduction

Leukocyte extravasation is a key feature of inflammation, and forms a critical component of host defence and tissue repair in response to injury and infection. Extensive investigation of the mechanisms of leukocyte diapedesis both *in vitro* and *in vivo* has unravelled many of the molecular and cellular pathways that mediate this crucial physiological process 1, 2. In addition to critical protective and regenerative roles, excessive or inappropriate inflammation can contribute to the development and progression of acute and chronic disorders as diverse as tumour growth 3, multiple sclerosis 4, arthritis 5, atherosclerosis 6, acute ischaemia–reperfusion (IR) injury 7, and chronic ischaemia associated with peripheral arterial disease (PAD) 8 or myocardial infarction 9.

Chronically inflamed tissues show biochemical and environmental abnormalities, including hypoxia, extracellular matrix deposition, angiogenesis, and, by definition, the presence of persistent populations of leukocyte infiltrates and/or ongoing recruitment of inflammatory cells. Chronic ischaemia of myocardial or skeletal muscle induces recruitment of both neutrophils and monocytes, with the latter differentiating into macrophages. The functions, properties and phenotype of tissue macrophages have been the subject of intense investigations, and these cells are broadly accepted as initially having proinflammatory, proteolytic and phagocytic functions, followed by roles supporting angiogenesis and tissue repair 9, 10, 11, 12, 13. Tissue macrophages regulate the recruitment of immune cells, and perturbation of resident populations is therefore likely to influence inflammatory responses within the tissue 9, 14, 15.

Much of our understanding of the mechanisms and dynamics of leukocyte recruitment stem from *in vivo* investigations carried out in physiologically normal tissues such as the mesentery, dermis, or cremaster muscle 14, 16, 17. However, the findings of such studies cannot be extrapolated precisely to events within the complex environment of chronically inflamed tissues. Direct investigation of mechanisms of leukocyte infiltration in chronic inflammatory conditions has been constrained by the fact that many experimental disease models do not allow easy visual access to the local vasculature, limiting analysis of vessel structures and dynamic inflammatory responses. The aim of this study was to employ a novel model of chronic ischaemia, inflammation and angiogenesis in the mouse cremaster muscle as a means of investigating how the cellular, molecular and vascular changes associated with chronic inflammation influence the profile, dynamics and mechanisms of acute inflammatory responses.

We observed that, in chronically inflamed post‐ischaemic (PI) tissues, large populations of proangiogenic Gr1^low^ monocyte‐derived cells (MDCs) substantially elevate acute neutrophil recruitment through the generation of proinflammatory mediators in response to acute pharmacological or physiological stimulation. These findings demonstrate that, in chronically inflamed tissues, both the mechanisms and the magnitude of acute inflammatory responses are altered, potentially exacerbating and prolonging tissue inflammation and adversely affecting healing. These findings have important implications for the interpretation of data obtained in healthy tissue models and the treatment of chronic inflammatory disorders.

## Materials and methods

Detailed materials and methods are available in the supplementary material, Supplementary materials and methods.

### Animals

Wild‐type (WT) C57BL/6 mice were purchased from Charles River Laboratories (Cambridge, UK). Heterozygous mice were used in which the gene for green fluorescent protein (GFP) has been knocked into the Cx3cr1 locus (*Cx3cr1‐GFP*) 18, or the lysozyme‐M locus (*LysM‐GFP*) 19, resulting in GFP expression in monocytes and neutrophils, respectively. All animal studies were approved of and performed within the UK Home Office Regulations on Animal Experimentation and our Institute's internal Ethical Review Process.

### Chronic ischaemia

Ischaemia and angiogenesis were induced in the mouse cremaster muscle or hindlimb by cauterization and/or excision of the main arteriole and vein perfusing the tissue 20, 21.

### Acute inflammatory responses

Acute inflammation of the cremaster or hindlimb muscles was induced by intrascrotal (i.s.), intramuscular (i.m.) or topical application of inflammatory stimuli.

### Immunofluorescence confocal microscopy

Confocal microscopy was used to analyse the extent and characteristics of vascularization and the frequency, distribution and morphology of different leukocyte subsets.

### Flow cytometry

Flow cytometry was used to analyse the frequency and phenotype of leukocyte populations in blood or enzymatically digested tissues, or to purify specific leukocyte subsets.

### Cell transfer model

Cx3cr1‐GFP^pos^ cells were purified from PI tissues and injected into naive hindlimb muscles. Acute IR of the hindlimb was induced by transient occlusion of the femoral artery and vein. Tissue leukocyte populations were then analysed by flow cytometry.

### Cytokine/chemokine array

The profile of cytokines and chemokines in muscle homogenates was analysed with an R&D Systems (Minneapolis, USA) mouse chemokine/cytokine array, according to the manufacturer's instructions.

### Reverse transcription– quantitative PCR (RT‐qPCR)

Levels of mRNAs for various inflammatory mediators were quantified by RT‐qPCR analysis of leukocyte subsets purified from digested cremaster muscles.

### Statistical analysis

Results are presented as mean ± standard error of the mean (SEM). Statistical significance was assessed with Student's *t*‐tests or one‐way anova with the Newman–Keuls multiple comparison test. Values of *p* < 0.05 were considered to be significant.

## Results

Prolonged ischaemia induces acute and chronic inflammatory responses and angiogenesis in the cremaster muscle

**Figure 1:**
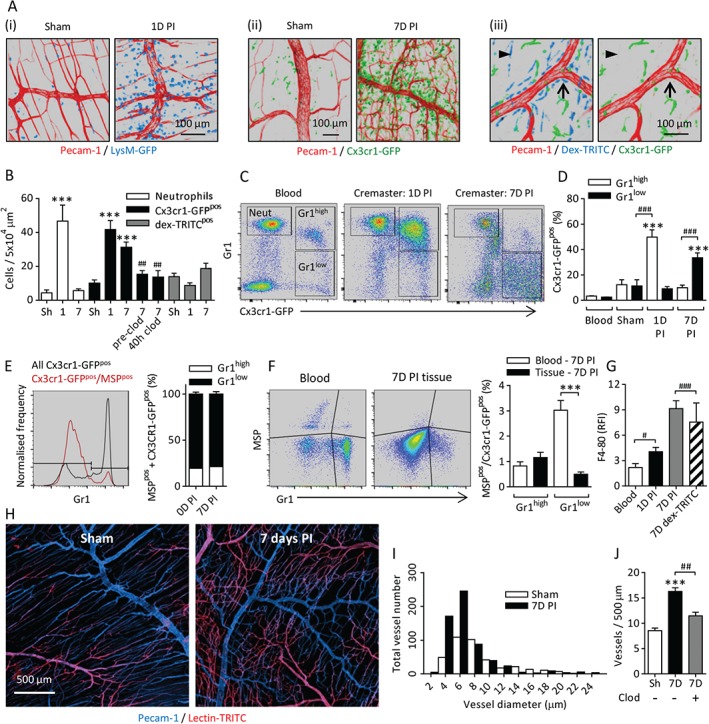
Prolonged ischaemia induces acute and chronic inflammatory responses and angiogenesis. Chronic ischaemia was induced in cremaster muscles by cauterizing the primary vessels perfusing the tissue. (A) Leukocyte populations were analysed in sham tissues and at 1 or 7 days PI with (i) LysM‐GFP and (ii) Cx3cr1‐GFP mice to visualize neutrophils and monocytes, respectively. (iii) Topical application (intrascrotal) of fluorescent dextran‐TRITC (dex‐TRITC) was used to visualize phagocytic cells in CX3CR‐1GFP^pos^ mice. The images show dex‐TRITC^pos^/Cx3cr1‐GFP^neg^ cells in perivascular (arrow) and non‐perivascular (arrowhead) locations in naive tissues. The vasculature was fluorescently labelled with anti‐PECAM‐1 antibody. (B) Quantification of neutrophils, Cx3cr1‐GFP^pos^ and Cx3cr1‐GFP^neg^/dex‐TRITC^pos^ cells in sham (Sh), 1‐day or 7‐day PI tissues. In some cases, circulating monocytes were depleted with clodronate (clod) liposomes (intravenous) before or 40 h after surgery. (C) Blood and cremaster muscles were collected from naive and 1‐day or 7‐day PI mice. Cremaster muscles were enzymatically digested, and cells were fluorescently labelled with antibodies against CD45 and Gr1 (clone RB6‐8C5) and 4′,6‐diamidino‐2‐phenylindole (DAPI) to identify live leukocytes (supplementary material, Figure S2i). Examples of leukocyte populations from blood and digested cremasteric tissues are shown. (D) Frequency of Cx3cr1‐GFP^pos^/Gr1^high^ or Gr1^low^ cells as a proportion of total CD45^pos^ leukocytes. (E) Analysis of blood after intravenous injection of fluorescent MSP. The histogram shows the frequencies of Gr1^high^ and Gr1^low^ monocytes in the circulation (black line), and the normalized frequency of MSP‐labelled monocytes. The graph shows Gr1^high^ or Gr1^low^ expression among all Cx3cr1‐GFP^pos^/MSP^pos^ cells. (F) Example scatterplots and quantification of MSP labelling of Cx3cr1‐GFP^pos^ cells in blood and 7‐day PI tissue after digestion. The graph shows the frequencies of MSP^pos^ Gr1^high^ and Gr1^low^ cells in blood and tissues 7 days PI (see supplementary material, Figure S2ii, for the full gating strategy) (G) F4/80 expression on Cx3cr1‐GFP^pos^/Gr1^low^ and dex‐TRITC^pos^ cells in blood and cremaster digests at 1 and 7 days PI, quantified relative to an isotype control (supplementary material, Figure S2i, iii). (H) Examples of sham or 7‐day PI tissues labelled with topical anti‐PECAM‐1 antibody to visualize venules and capillaries, with less intense labelling of arterioles, and lectin‐TRITC (intravenous) to visualize arterial vessels and capillaries, with less intense labelling of venules. (I) Frequency histogram of vessel diameters in 12 images of sham and PI tissues. (J) Circulating monocytes were depleted with clodronate liposomes (intravenous) prior to surgery. The frequency of vessels was quantified in sham, PI and monocyte‐depleted 7‐day PI tissues. n = 5–15 animals per group. Data are presented as mean ± SEM, and statistically significant (t‐test) differences between sham and PI groups or blood and tissues are indicated by asterisks: *p < 0.05, **p < 0.01, and ***p < 0.001. Differences between control and monocyte‐depleted groups, cell subsets or time points are indicated by hash symbols: ^#^ p < 0.05, ^##^ p < 0.01, and ^###^ p < 0.001.

Chronic ischaemia was induced in murine cremaster muscles by cauterization of the main vessels supplying the tissue. Intravenous fluorescent microbeads and the hypoxia probe pimonidazole demonstrated that this procedure induces partial ischaemia and hypoxia (supplementary material, Figure S1A, B). The presence of neutrophils, monocytes and macrophages in sham and PI tissues was investigated in LysM‐GFP and Cx3cr1‐GFP mice 18, 19, in conjunction with locally applied fluorescent dextran‐TRITC (i.s.) to visualize phagocytic cells. Sham tissues showed low numbers of neutrophils and pronounced infiltration at 1 day PI, which had resolved by 7 days PI (Figure 1Ai, B). A population of Cx3cr1‐GFP^pos^ cells was present in naive and sham tissues, and was significantly greater at 1 day PI, with this elevation persisting at 7 days PI. Clodronate liposomes were administered intravenously to deplete circulating monocytes before surgery, or 40 h after surgery. This procedure abolished the elevation in tissue‐resident Cx3cr1‐GFP^pos^ cells at day 7, confirming that this population derives from circulating monocytes rather than *in situ* proliferation of the homeostatic tissue‐resident population (Figure 1Aii, B). Note that intravenous liposomes do not pass through the endothelial barrier and do not affect tissue‐resident cells (not shown). Cells with a high level of dextran‐TRITC uptake but no Cx3cr1‐GFP expression (dextranTRITC^pos^/Cx3cr1‐GFP^neg^) were seen throughout the tissues, and were particularly prevalent in perivascular locations. The frequency of these cells did not change after induction of ischaemia (Figure 1Aiii, B). These cells were morphologically similar to perivascular macrophages described previously 14.

The phenotype of Cx3cr1‐GFP^pos^ cells was analysed by flow cytometry (see supplementary material, Figure S2, for the full gating strategy). Expresssion of Gr1 (also known as Ly6G/Ly6C) detected with the RB6‐8C5 monoclonal antibody, along with Cx3cr1‐GFP expression in monocytes, was used to identify neutrophils and the two major monocyte subsets (Cx3cr1‐GFP^pos^/Gr1^high^ or Gr1^low^) (Figure 1C). PI cremaster muscles had more Cx3cr1‐GFP^pos^ cells; these were initially Cx3cr1‐GFP^pos^/Gr1^high^, but, at later times, Cx3cr1‐GFP^pos^/Gr1^low^ cells were predominant (Figure 1C, D).

In order to determine whether Cx3cr1‐GFP^pos^/Gr1^high^ cells downregulate Gr1 *in situ*, or whether there is later recruitment of Cx3cr1‐GFP^pos^/Gr1^low^ cells, we used a model in which intravenously delivered fluorescent microspheres (MSPs) are phagocytosed by, and so selectively label, Gr1^low^ monocytes in the circulation, without affecting recruitment to inflamed tissues 22, 23, 24. MSPs were administered 48 h prior to surgery. At the time of surgery 1.7 ± 0.8% Gr1^high^ monocytes were MSP^pos^, and this proportion had fallen to 0.8 ± 0.4% by 7 days post‐surgery. However, Gr1^low^ monocytes were 7.9 ± 0.47% and 3.0 ± 0.9% MSP^pos^ at these time points, demonstrating that, throughout the post‐surgical period, ∼80% of MSP^pos^ monocytes in the circulation were Gr1^low^ (Figure 1E; supplementary material, Figure S2ii). The low frequency of Gr1^high^/MSP^pos^ cells was comparable in the blood and the 7‐day PI tissues, whereas the frequency of Gr1^low^/MSP^pos^ cells was significantly lower in the tissue than in the circulation (Figure 1F; supplementary material, Figure S2ii). These data indicate that Cx3cr1‐GFP^pos^/Gr1^low^ cells are not recruited to the tissues in substantial numbers, and that *in situ* downregulation of Gr1 is the most likely explanation for the phenotypic differences in Cx3cr1‐GFP^pos^ cells observed between 1‐day and 7‐day PI tissues.

Cx3cr1‐GFP^pos^/Gr1^low^ cells also showed increased expression of the macrophage marker F4‐80 at 1 day PI, and this was further elevated at 7 days PI. Dextran‐TRITC^pos^/Cx3cr1‐GFP^neg^ cells also expressed F4/80, confirming their macrophage lineage (Figure 1G; supplementary material, Figure S2i, iii). Cx3cr1‐GFP^pos^/Gr1^low^ cells showed no change in F4/80 expression between 1 day PI and 7 days PI [relative fluorescence intensity (RFI); 3.7 ± 1.5 and 5.0 ± 2.3, respectively]. Cx3cr1‐GFP^pos^ cell morphology was altered between 1 and 7 days PI (supplementary material, Figure S1C). It is of note that T cells and natural killer (NK) cells have been reported to express Cx3cr1 18, 25, but very few CD3‐expressing T cells or CD335‐expressing NK cells were seen in 7‐day PI tissues, further indicating that this population is of a monocyte/macrophage lineage (supplementary material, Figure S2iv).

The effect of chronic ischaemia and inflammation on the cremasteric vasculature was investigated. Sham tissues showed an organized vascular tree in which capillary networks efficiently deliver oxygenated blood to the regions between arterioles and venules, whereas, at 7 days PI, the capillary networks appeared denser and more disorganized (see Figure 1H and supplementary material, Figure S1D, for more examples). The majority (>90%) of vessels in both the sham and 7‐day PI tissues had a functional lumen (supplementary material, Figure S1Ei–iii).

PI tissues had an increased frequency of small‐diameter capillary‐like vessels, but no changes in the frequency or gross morphology of larger‐diameter venules or arterioles were detected (Figure 1I). Depletion of circulating monocytes with intravenous clodronate liposomes prior to surgery and subsequent prevention of recruitment to PI tissues inhibited the angiogenic response to chronic ischaemia (Figure 1J).

Collectively, these data demonstrate that an influx of neutrophils and Gr1^high^ monocytes from the blood occurs rapidly after induction of ischaemia, and ongoing recruitment of Gr1^high^ monocytes may also occur. Following phenotypic changes *in situ*, Gr1^low^ MDCs with elongated morphology and elevated F4/80 expression persist in the tissue for a prolonged period of time after the initial injury. This population of MDCs supports the proliferation of small‐diameter capillary‐like vessels with a functional lumen. Thus the cremaster muscle shows a similar profile of inflammatory and angiogenic responses to that seen in other animal models of chronic ischaemia 26, 27, but is significantly more amenable to high‐resolution imaging.

### Chronically inflamed PI tissues show elevated acute neutrophil recruitment

**Figure 2:**
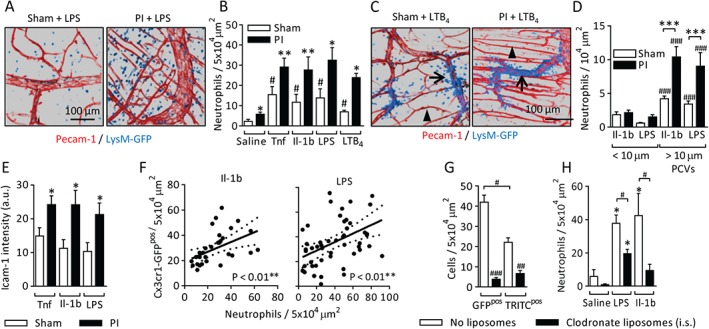
PI tissues show elevated acute neutrophil recruitment. Chronic ischaemia was induced in the cremaster muscles of LysM‐GFP mice, which have GFP^pos^ neutrophils. After 7 days, the tissues were fluorescently labelled with antibodies against PECAM‐1 (i.s.) and stimulated with TNF (300 ng), IL‐1β (50 ng), LPS (300 ng) or LTB_4_ (100 nm, i.s.) 4 h before imaging. (A) Examples of LPS‐stimulated sham and PI tissues. (B) Quantification of neutrophil extravasation. (C) Still images from supplementary material Videos S1 and S2, showing neutrophil extravasation in sham and PI tissues after stimulation with topical LTB_4_ (100 nm). Examples of postcapillary venules (PCVs) (arrows) and capillaries (arrowheads) are indicated. (D) Quantification of luminal neutrophil adhesion in small‐diameter capillary‐like vessels (<10 µm) and larger PCVs (>10 µm) 2 h after stimulation with TNF, IL‐1β, or LPS (i.s.). The data are normalized to luminal surface area. (E) Endothelial ICAM‐1 labelling intensity in PCVs in sham or 7‐day PI tissues (see supplementary material, Figure S1G, for images). (F) At 7 days PI, Cx3cr1‐GFP tissues were acutely stimulated with IL‐1β (50 ng) or LPS (300 ng) (i.s., 4 h), collected, and fluorescently labelled with antibodies against PECAM‐1 and the neutrophil marker S100A9. Correlations of the spatial distributions of Cx3cr1‐GFP^pos^ cells and neutrophils in 5 × 10^4^‐µm^2^ regions of IL‐1β‐stimulated (left panel) or LPS‐stimulated (right panel) tissues (see supplementary material, Figure S1H, for an example image) are shown. (G) Depletion of Cx3cr1‐GFP^pos^ and Cx3cr1‐GFP^neg^/dextran‐TRITC^pos^ cells by local application (i.s.) of clodronate liposomes on days 6–8 PI. (H) LPS‐induced or IL‐1β‐induced neutrophil recruitment in control or monocyte/macrophage‐depleted tissues. n = 6–12 animals per group. Data are presented as mean ± SEM, and statistically significant (t‐test) differences between sham and PI groups or correlations are indicated by asterisks: *p < 0.05, **p < 0.01, and ***p < 0.001. Differences between saline and stimulated groups, vessel size groups or control and clodronate‐depleted groups are indicated by hash symbols: ^#^ p < 0.05, ^##^ p < 0.01 and ^###^ p < 0.001.

A primary aim of this study was to compare the profile and mechanisms of acute inflammatory responses in chronically inflamed ischaemic tissues with those of physiologically normal tissues. Seven‐day PI cremaster muscles of LysM‐GFP^pos^ mice were acutely stimulated with interleukin (IL)‐1β, tumour necrosis factor (TNF), lipopolysaccharide (LPS), leukotriene B4 (LTB_4_), or saline, and neutrophil infiltration was quantified. PI tissues showed substantially greater neutrophil influx than sham tissues in each stimulus group (Figure 2A, B).

Intravital confocal microscopy was used to investigate the route of neutrophil extravasation. PECAM‐1‐labelled sham or PI tissues were exteriorized, LTB_4_ was applied topically, and the inflammatory response was imaged. There was extravasation from postcapillary venules, but not from capillaries, in both sham and PI tissues (Figure 2C; supplementary material, Videos S1 and S2). The vascular location of luminally adherent neutrophils prior to extravasation demonstrated that little adhesion occurs in vessels of diameter <10 µm, and that, as in sham tissues, postcapillary venules are the primary site of adhesion in PI tissues, with significantly higher levels of luminal adhesion than in sham tissues (Figure 2D). Elevated levels of endothelial ICAM‐1 expression were seen in stimulated PI tissues, suggesting that endothelial activation contributes to elevated neutrophil adhesion and recruitment (Figure 2E; supplementary material, Figure S1F).

As neovascularization did not account for elevated neutrophil influx, we investigated the possibility that the increased responses were related to the abundant population of MDCs present. Sham or PI cremaster muscles of Cx3cr1‐GFP mice were stimulated with IL‐1β or LPS, and labelled with fluorescent antibodies against PECAM‐1 and the neutrophil marker S100A9. A correlation between areas with abundant Cx3cr1‐GFP^pos^ cells and high levels of neutrophil recruitment was seen (Figure 2F; supplementary material, Figure S1G). When tissue‐resident monocytes and macrophages were depleted by locally applied clodronate liposomes (i.s.) on day 6, after influx of blood monocytes and vessel proliferation had taken place (Figure 2G), IL‐1β‐induced or LPS‐induced neutrophil recruitment was significantly reduced (Figure 2H).

These data demonstrate that, in PI tissues, morphologically normal postcapillary venules show greater levels of stimulation‐dependent ICAM‐1 expression, luminal neutrophil adhesion, and extravasation. An association between MDCs in chronically inflamed tissues and elevated acute inflammatory responses was also seen.

### Acute stimulation of Gr1^low^ MDCs induces generation of proinflammatory signals

**Figure 3:**
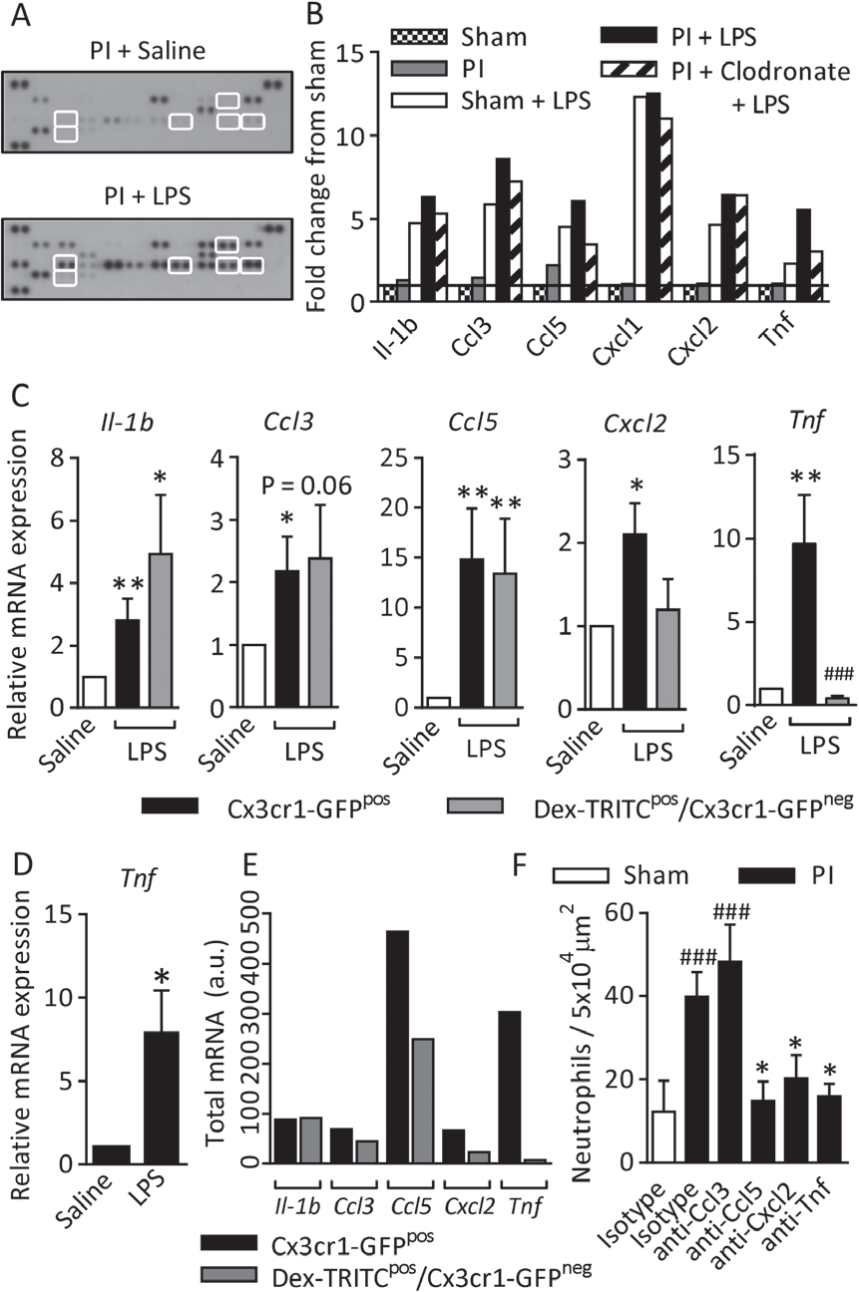
Acute LPS stimulation of Gr1^low^ MDCs induces the generation of proinflammatory signals. Chronic ischaemia was induced in the cremaster muscles of WT mice. Some tissues were monocyte/macrophage‐depleted with clodronate liposomes (i.s.), and circulating neutrophils were depleted with anti‐Ly6G antibody (clone 1A8). On day 7, cremaster muscles were acutely stimulated with LPS (300 ng, i.s., 4 h) or saline. Tissues were collected, homogenized, and analysed by use of a chemokine/cytokine array immunoblot. (A) Examples of an unstimulated blot and an LPS‐stimulated blot. Highlighted spots are the mediators shown in (B). (B) Intensity data were analysed for mediators that conformed to certain parameters: (1) known to recruit neutrophils; (2) elevated by LPS stimulation; (3) higher levels in PI than in sham tissues; and (4) reduced in monocyte/macrophage‐depleted tissues. Data are normalized to the fold difference from unstimulated sham tissues. n = 2 animals pooled per blot, two blots (n = 4 animals) per treatment group (see supplementary material, Table S1, for the complete dataset). (C) Seven‐day PI cremaster muscles from Cx3cr1‐GFP mice were stimulated with LPS or saline, and phagocytic cells were labelled with TRITC‐dextran (i.s.) in vivo. Tissues were digested enzymatically, and labelled with anti‐CD45 and 4′,6‐diamidino‐2‐phenylindole (DAPI) to identify live leukocytes. CD45^pos^/DAPI^neg^/Cx3cr1‐GFP^pos^ cells (which have a variable level of dextran‐TRITC intensity) and DAPI^neg^/CD45^pos^/dextran‐TRITC^pos^/Cx3cr1‐GFP^neg^ cells were purified by fluorescence‐activated cell sorting (see supplementary material, Figure S2, for the full gating strategy). Purified cell populations were analysed by RT‐qPCR for Il‐1b, Ccl3, Ccl5, Cxcl2 and Tnf mRNA. Data were normalized to the glyceraldehyde‐3‐phosphate dehydrogenase gene, and are shown as the fold difference between LPS‐stimulated cells (black and grey bars) and unstimulated samples of each cell type (white bars). (D) The Cx3cr1‐GFP^pos^/Gr1^low^ population was selectively purified from saline and LPS‐stimulated tissues, and Tnf mRNA was quantified. (E) The frequency of dextran‐TRITC^pos^/Cx3cr1‐GFP^neg^ and Cx3cr1‐GFP^pos^ cells (from Figure 1B) was multiplied by the expression level of each mediator to visualize the total inflammatory contribution of each cell population. (F) Sham or 7‐day PI tissues were treated with blocking antibodies against Ccl3, Ccl5, Cxcl2 or TNF (10–20 µg) co‐administered with LPS (300 ng, i.s., 4 h). Tissues were collected and labelled with fluorescent antibodies against PECAM‐1 and S100A9, and neutrophil extravasation was quantified. n = 3–11 animals per group. Data are presented as mean ± SEM, and statistically significant (t‐test) differences between saline and LPS‐stimulated groups, or between isotype control and blocking antibody groups, are indicated by asterisks: *p < 0.05, **p < 0.01, and ***p < 0.001. Differences between sham and PI tissues or between Cx3cr1‐GFP^pos^ cells and dextran‐TRITC^pos^/Cx3cr1‐GFP^neg^ cells are indicated by hash symbols: ^#^ p < 0.05.

We hypothesized that acute stimulation of MDCs produces additional inflammatory mediators, amplifying the original stimulus and elevating neutrophil recruitment. Thus, it should be possible to identify known neutrophil stimuli that are: (1) elevated by LPS stimulation; (2) present at higher levels in stimulated PI tissues than stimulated sham tissues, and (3) are present at lower levels if the tissues are depleted of monocytes/macrophages prior to stimulation. The profile of inflammatory mediators generated following acute LPS stimulation of sham tissues, PI tissues and tissues depleted of monocytes/macrophages was analysed in whole tissue homogenates by use of a chemokine/cytokine array. Examples of unstimulated and LPS‐treated PI tissue blots and the intensity values for all mediators are shown in Figure 3A (see supplementary material, Table S1, for complete data). With application of the criteria described above, several candidate mediators were identified: IL‐1β, Ccl3, Ccl5, Cxcl1, Cxcl2, and TNF (Figure 3B).

Changes in mRNA levels of candidate mediators specifically by Cx3cr1‐GFP^pos^ and dextran‐TRITC^pos^/Cx3cr1‐GFP^neg^ cells purified from control and LPS‐stimulated PI tissues was analysed by RT‐qPCR (see supplementary material, Figure S2iii, for gating). The abundance in unstimulated cells was normalized to 1, and values from LPS‐stimulated cells of each phenotype were expressed as fold difference (Figure 3C) 28. *Il‐1b*, *Ccl3* and *Ccl5* mRNAs were upregulated in both cell types, whereas *Cxcl2* and *Tnf* were upregulated only in Cx3cr1‐GFP^pos^ cells. *Cxcl1* was not upregulated by either of the cell populations examined (data not shown). The majority (∼70%) of Cx3cr1‐GFP^pos^ cells in 7‐day PI tissues showed a Gr1^low^ phenotype, but specific analysis of the Gr1^low^ population demonstrated that the more abundant Gr1^low^ subset upregulated *Tnf* mRNA levels to a similar degree, and thus make a significant contribution to the proinflammatory response in these tissues (Figure 3D). In order to illustrate the relative inflammatory contributions of Cx3cr1‐GFP^pos^ and dextran‐TRITC^pos^/Cx3cr1‐GFP^neg^ cells, we multiplied the frequency of each cell type (Figure 1B) by the mRNA level of each mediator to generate a measure of the total mRNA generation in LPS‐stimulated 7‐day PI tissues (Figure 1E). The functional role of mediators generated by tissue‐resident cells was confirmed by inhibition of neutrophil recruitment by co‐application of neutralizing antibodies against Ccl5, Cxcl2 or TNF with the LPS stimulus. Anti‐Ccl3 had no inhibitory effect (Figure 3F).

Collectively, these data show that Cx3cr1‐GFP^pos^/Gr1^low^ MDCs contribute to elevated neutrophil recruitment by altering the profile and magnitude of secondary signal generation. These mechanisms are summarized in Figure 6.

### Ischaemic hindlimb muscles show vascular and inflammatory profiles similar to those of the cremaster muscle

**Figure 4:**
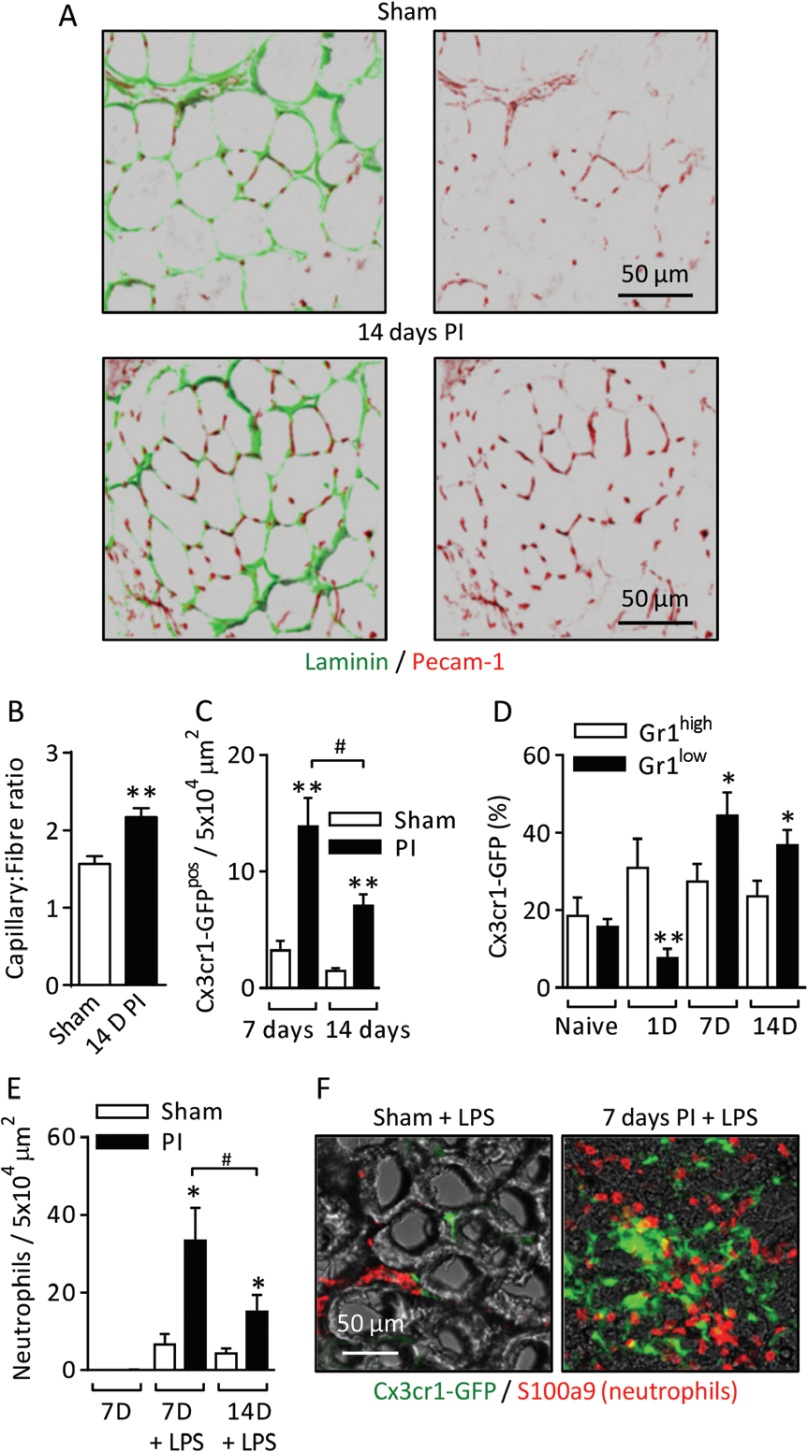
Ischaemic hindlimb muscles show vascular and inflammatory profiles similar to those of the cremaster muscle. Chronic ischaemia was induced in the hindlimbs of WT, Cx3cr1‐GFP or LysM‐GFP mice by occlusion of the femoral artery and vein. The anterior tibialis muscles from sham or PI limbs were collected at 7 or 14 days after surgery, and either examined as whole mount muscles, or fixed and sectioned for labelling with fluorescent antibodies against laminin, PECAM‐1, or S100A9. (A) Example images of capillaries (PECAM‐1) and muscle fibres (laminin) in sham and 14‐day PI tissue sections. (B) Quantification of the capillary/fibre ratio in sham and 14‐day PI muscles. (C) The frequency of Cx3cr1‐GFP^pos^ cells was quantified in whole mount tissues at 7 and 14 days PI. (D) Muscles were collected from naive and 1‐day, 7‐day or 14‐day PI mice, digested enzymatically, and labelled fluorescently with antibodies against CD45 and Gr1 (clone RB6‐8C5) and 4′,6‐diamidino‐2‐phenylindole (DAPI) to identify live leukocytes (see supplementary material, Figure S2, for the full gating strategy). The frequency of Cx3cr1‐GFP^pos^/Gr1^high^ or Gr1^low^ cells as a proportion of total CD45^pos^ leukocytes is shown. (E) In experiments with LysM‐GFP mice, PI or sham anterior tibialis muscles were injected with LPS (100 ng, 4 h) at 7 or 14 days PI. Neutrophil recruitment was quantified in whole mount tissues. (F) Example images of Cx3cr1‐GFP^pos^ cells and S100A9^pos^ neutrophils in LPS‐stimulated sham and 7‐day PI tissue sections. n = 6–8 animals per group. Data are presented as mean ± SEM, and statistically significant (t‐test) differences between sham and PI groups are indicated by asterisks: *p < 0.05, **p < 0.01, and ***p < 0.001. Differences between time points are indicated by hash symbols: ^#^ p < 0.05.

The mouse femoral artery occlusion model is used widely in the study of PAD, and was employed here to examine the relationship between chronic ischaemia, angiogenesis, MDCs and acute neutrophil recruitment in a clinically relevant pathophysiological model. Induction of chronic ischaemia induced an increase in the capillary/muscle fibre ratio by 14 days PI (Figure 4A, B). More Cx3cr1‐GFP^pos^ cells were seen in PI muscles at 7 and 14 days PI (Figure 4C, F). A similar pattern of Gr1^high^ cells at early time points, with Gr1^low^ cells predominant at 7 and 14 days PI, was also seen (Figure 4D). Importantly LPS (intramuscular)‐stimulated recruitment of neutrophils was significantly increased in 7‐day and 14‐day PI tissues (Figure 4E, F). Thus, similar vascular and inflammatory responses are seen in the ischaemic hindlimb and the cremaster muscle.

### Gr1^low^ MDCs amplify the response to acute ischaemia and reperfusion

PAD patients commonly experience exercise‐induced pain, known as claudication, as a result of insufficient perfusion and oxygenation of tissues 29. To further investigate the clinical relevance of proinflammatory MDCs in chronically inflamed tissues, we used a model of acute IR of the hindlimb, intended to mimic transient exercise‐induced ischaemia, in conjunction with cell transfer of MDCs.

**Figure 5:**
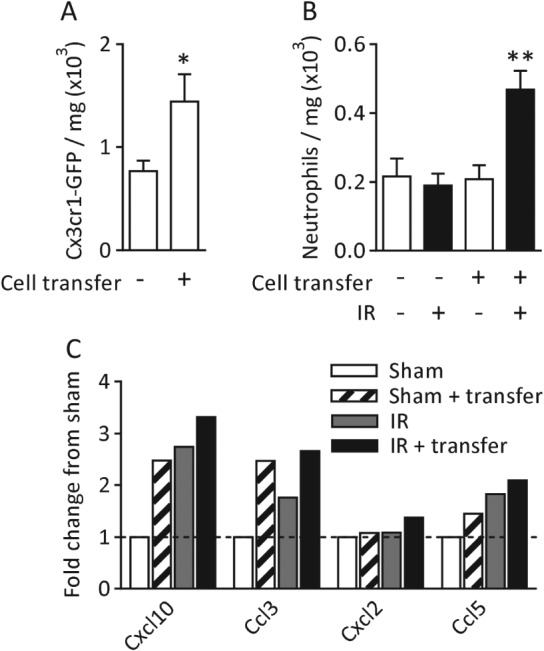
Gr1^low^ MDCs amplify the response to acute IR. (A) Cx3cr1‐GFP^pos^ cells were counted in naive tissues and in those that received cell transfer. (B) Quantification of neutrophils in naive, cell transfer and cell transfer with IR tissues. (C) Quantification of chemokines and cytokines in whole tissue homogenates (see supplementary material, Table S2, for the complete dataset). Values are shown as the fold‐difference from sham. n = 3–6 animals per group for flow cytometry, and pooled tissues from six animals for the cytokine/chemokine array. Data are presented as mean ± SEM, and statistically significant (t‐test) differences between groups are indicated by asterisks: *p < 0.05 and **p < 0.01.

Cx3cr1‐GFP^pos^ cells were isolated from 7‐day PI cremaster muscles and injected into the hindlimb muscle of naive Cx3cr1‐GFP mice. Acute ischaemia (60 min) and reperfusion (120 min) of a hindlimb were induced, and the leukocyte profile of the tissues was analysed by flow cytometry (see supplementary material, Figure S3, for the cell transfer method). Cell transfer approximately doubled the total number of Cx3cr1‐GFP^pos^ cells present in the muscle (Figure 5A). Whereas IR or cell transfer alone did not induce neutrophil recruitment, the combination of cell transfer and IR did induce significant neutrophil recruitment (Figure 5B). The profile of inflammatory mediators in these tissues was analysed by use of a chemokine/cytokine array (see supplementary material, Table S2, for complete data), and several neutrophil stimuli were found to be elevated in response to cell transfer, IR, or cell transfer and IR combined (Figure 5C).

These data indicate that tissue‐resident MDCs from chronically inflamed tissues have the potential to respond to transient IR by the release and/or *de novo* generation of inflammatory mediators, thus inducing neutrophil recruitment in response to otherwise subthreshold IR events.

## Discussion

**Figure 6:**
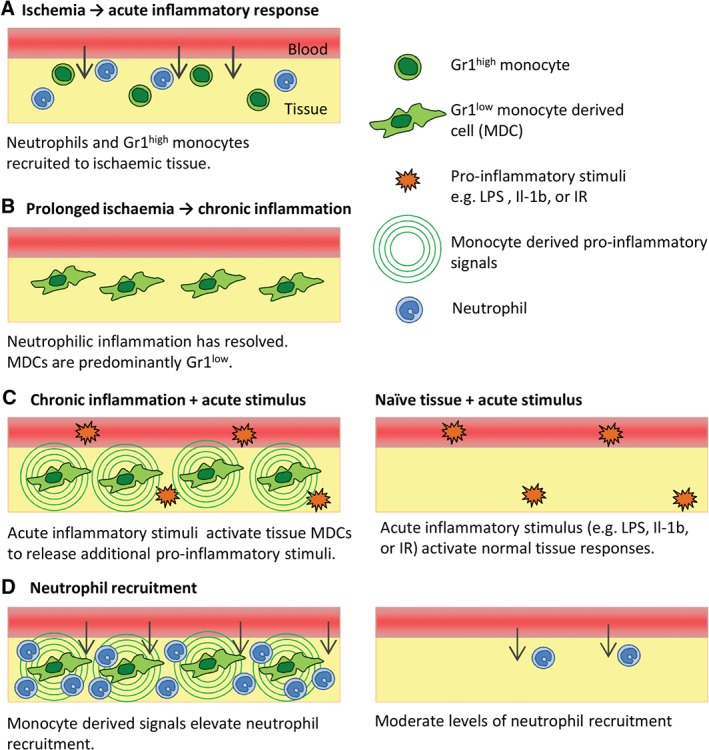
Schematic illustration of the interaction of acute and chronic inflammatory responses. (A) Induction of chronic partial ischaemia induces rapid recruitment of neutrophils and Gr1^high^ monocytes from the circulation. (B) Seven days after the induction of ischaemia, the neutrophilic inflammation has resolved, but an elevated population of Gr1^low^ MDCs persist in the tissue. (C) These Gr1^low^ MDCs are highly sensitive to a range of pharmacological or physiological stimuli (e.g. LPS or IR), and respond by generating a range of additional proinflammatory stimuli. (D) These secondary signals act on the endothelium to upregulate ICAM‐1 expression, which supports luminal neutrophil adhesion, and/or directly activate neutrophil chemotaxis. Naive tissues exposed to the same exogenous stimuli lack this additional source of secondary mediators, resulting in lower overall tissue activation.

The mechanisms governing leukocyte extravasation have been studied extensively. In the majority of *in vivo* models employed, acute inflammatory responses are induced in otherwise healthy tissues 14, 16, 17; however, in many clinical conditions, chronic inflammation and attendant tissue abnormalities are prominent features, and it is likely that the responses in these tissues will be mediated by very different mechanisms. Here, we have investigated the mechanisms governing acute leukocyte extravasation in chronically inflamed and angiogenic tissues as compared with physiologically normal conditions. Our findings describe a population of ischaemia‐associated and angiogenesis‐associated cells that are derived from circulating monocytes, and can amplify modest inflammatory insults through the generation of additional proinflammatory signals (Figure 6). This makes the tissues highly sensitive to acute inflammatory stimuli, and may affect disease progression and the resolution of chronic inflammation.

In a novel model of permanent occlusion of the main vessels supplying the mouse cremaster muscle, chronic partial ischaemia and hypoxia were induced, triggering acute recruitment of neutrophils and Gr1^high^ monocytes. A population of Gr1^low^ MDCs then persisted for at least 7 days. Other populations of tissue‐resident macrophages were identified by their ability to phagocytose dextran‐TRITC, F4/80 expression, and their morphological similarity to previously identified perivascular macrophages 14. This abundance of this population was not altered in ischaemic tissues.

These biphasic populations of Gr1^high^ and Gr1^low^ monocytes have previously been reported 24, 26, 27, 30. Some reports have shown that Gr1^high^ monocytes downregulate Gr1 *in situ* 24, whereas others have reported that a second wave of Cx3cl1‐dependent recruitment of Gr1^low^ monocytes from the blood subsequently occurs 26, 27. In our model, depletion of circulating monocytes 40 h after surgery (by which time an initial influx of monocytes to the tissue had occurred) inhibited the increase in the number of tissue‐resident cells at day 7, suggesting that there is ongoing recruitment for some time after the initial injury. Specific labelling of Gr1^low^ monocytes in the circulation indicated that, in this model, there is not substantial recruitment of Gr1^low^ monocytes from the blood to the ischaemic tissue, although, as only a small percentage of circulating Gr1^low^ monocytes were labelled with fluorescent microspheres, we cannot totally exclude the possibility that the labelled Gr1^low^ cells represent a less transmigratory subset than the unlabelled cells. Taken together, these data suggest that ongoing recruitment of Gr1^high^ monocytes followed by Gr1 downregulation *in situ* is likely to occur.

In line with previous studies, PI cremaster muscles showed monocyte‐dependent proliferation of capillary‐like vessels 26, 27, 30. Many animal models of chronic inflammation and angiogenesis, e.g. arthritic joints or large skeletal muscles, require histological sectioning or highly specialized techniques such as those employed in studies of inflammatory responses in plaque‐associated vasa vasorum 31. Permanent ligation of the cremasteric branch of the rat hypogastric artery has previously been shown to elevate the frequency of second‐order and third‐order arterioles after 6 weeks, presumably by the process of collateral artery remodelling, but no information on inflammatory responses was reported in these studies 32, 33. This model provides a powerful tool for high‐resolution imaging of inflammatory and angiogenic responses, and it is anticipated that this system will be of value to future studies of the mechanisms associated with inflammatory angiogenesis.

Although the link between chronic ischaemia and inflammation is well established, to our knowledge few studies have directly investigated how underlying chronic inflammation affects responses to acute inflammatory insults. In the present study, PI tissues consistently showed greater neutrophil recruitment than sham tissues, and this excessive recruitment of neutrophils could both contribute to tissue damage 34 and support ongoing angiogenesis 35, 36, 37. Several possible explanations for this increased recruitment were considered, including: (1) that the vasculature in PI tissues was structurally compromised, allowing more neutrophils to transmigrate; (2) that the increased vascularity of the PI tissues provided more opportunities for neutrophil egress; or (3) that the normal mechanisms of leukocyte recruitment are altered and/or highly activated in the PI tissues.

At the level of individual vessels, we did not observe any overt abnormalities in terms of PECAM‐1 or VE‐cadherin (not shown) expression in either the capillaries or larger‐diameter postcapillary venules and arterioles. Analysis of the site of luminal adhesion preceding extravasation, in conjunction with *in vivo* imaging, confirmed that pre‐existing postcapillary venules, rather than the capillary neovasculature, support extravasation in PI tissues. These venules showed greater stimulus‐dependent neutrophil adhesion and endothelial ICAM‐1 expression than in sham tissues, indicating that enhanced activation of the venular endothelium may contribute to elevated neutrophil recruitment 1, 2.

Postcapillary venules are the primary sites of leukocyte extravasation, and so it is perhaps not surprising that, in the already highly vascularized skeletal muscle used in this model, the capillary neovasculature did not act as a key route for diapedesis. In other pathophysiological scenarios in which the affected tissues are not well vascularized in the non‐disease state, e.g. arthritic joints or arterial walls, angiogenesis may indeed provide a novel route of leukocyte or plasma protein entry 31, 38, 39, 40, 41, 42.

Having determined that postcapillary venules are highly activated in PI tissues, we investigated a possible link with the abundant ischaemia‐associated MDCs, the distribution of which correlated with areas of high neutrophil recruitment. Depletion of tissue‐resident monocytes and macrophages prior to acute stimulation reduced subsequent neutrophil recruitment. Perivascular macrophages identified in the dermis and cremaster muscle have been shown to support neutrophil recruitment through the generation of inflammatory mediators 14, but the numbers, and hence likely contribution, of this population are not altered between sham and PI tissues in this model, and the Cx3cr1‐GFP^pos^ cells are more numerous in chronically inflamed tissues, thus generating a greater proportion of proinflammatory signals. Taken together, our findings suggest that MDCs associated with ischaemia and angiogenesis support elevated neutrophil recruitment in PI tissues.

Gr1^high^ monocytes have been reported to generate inflammatory mediators such as IL‐1β, TNF, and Ccl2, and to have a phagocytic and proteolytic role in the early response to injury 24, 27. In contrast, unstimulated Gr1^low^ cells express anti‐inflammatory signals such as IL‐10 and transforming growth factor‐β, and are important in tissue repair and revascularization 9, 24. Therefore, whereas Gr1^low^ MDCs show anti‐inflammatory and reparative characteristics under resting conditions, we have associated activation of these cells with the generation of a range of proinflammatory signals and elevated acute neutrophil recruitment. Neutralizing antibodies against some of these signals confirmed their functional role in LPS‐stimulated neutrophil recruitment. TNF and IL‐1β stimulate neutrophil recruitment via direct activation of neutrophils and increased endothelial ICAM‐1 expression 43, 44, and Cxcl2, Ccl3 and Ccl5 have been shown to recruit both neutrophils and monocytes 45, although we saw no effect of Ccl3 blockade. These data demonstrate that, in chronically inflamed tissues, not only is the response to an acute inflammatory stimulus substantially elevated, but the profile of secondary signals generated is altered.

To investigate the broader clinical relevance of these findings, we employed the murine femoral artery occlusion model, which is widely used in the study of PAD, despite some limitations when an acute vessel occlusion animal model is compared with the progressive arterial stenosis seen in patients 20, 21. The muscle of the lower hindlimb showed a similar profile to the cremaster muscle of capillary angiogenesis, tissue‐resident Cx3cr1‐GFP^pos^ cells, and enhanced neutrophil recruitment. PAD patients often experience exercise‐induced pain known as claudication, resulting from transient ischaemia/hypoxia of the affected limbs, which are unable to meet the increased oxygen demand during exercise 29.

By injecting PI tissue‐derived MDCs into naive limbs, which were then subjected to transient IR, we demonstrated that introduction of Cx3cr1‐GFP^pos^ cells from chronically inflamed tissues made the tissues sensitive to an IR stimulus that, on its own, was insufficient to induce a neutrophil migration response, possibly as a result of the abundant collateral arteries present in C57BL6 mice 46. This increased sensitivity to an apparently mild IR stimulus suggests that the transferred cells are phenotypically different and more responsive to IR than the homeostatic Cx3cr1‐GFP^pos^ tissue‐resident population.

The present study demonstrates that abundant Gr1^low^ MDCs in chronically inflamed tissues, which are phenotypically distinct from homeostatic tissue macrophage populations, significantly alter the profile and magnitude of responses to a wide range of pharmacological or physiological stimuli, making the tissue highly sensitive to acute inflammatory stimuli. These changes may, in turn, affect the severity, progression and duration of chronic inflammation, and these observations highlight the risks of extrapolating data obtained in studies of healthy tissues to pathological scenarios. Furthermore, our results suggest the real possibility of uncoupling beneficial effects of macrophages in terms of tissue revascularization and reperfusion 10, 47, 48 from potentially harmful proinflammatory effects. Collectively, the data from present study highlight the need for a better understanding of the profile, dynamics and mechanisms of inflammatory responses within chronically inflamed tissues, and strongly suggest that such studies may open opportunities for therapeutic interventions that could break the cycle of inflammation without having significant adverse effects on the host's normal immune or tissue repair responses.

## Author contributions statement

The authors contributed in the following way: BM: performed most experiments and analysed data; JRW: contributed to RT‐qPCR experiments; SN: contributed to the project design and critical reading of the manuscript, and provided facilities and equipment; AW: carried out experiments, provided overall project supervision, and produced the manuscript and figures.

**SUPPLEMENTARY MATERIAL ONLINE**

**Supplementary materials and methods**

**Supplementary figure legends**

**Figure S1.** Additional images and data.

**Figure S2.** Gating strategies for flow cytometry.

**Figure S3.** Cell transfer model.

**Table S1.** Intensity data for chemokine/cytokine immunoblot array of LPS‐stimulated cremasters.

**Table S2.** Intensity data for chemokine/cytokine immunoblot array from acute ischaemia/reperfusion‐stimulated hindlimbs.

**Video S1.** Neutrophil extravasation in sham tissues.

**Video S2.** Neutrophil extravasation in PI tissues.

## Supporting information


**Supplementary materials and methods**

**Supplementary figure legends**

**Figure S1. Additional images and data**. Chronic ischaemia was induced in cremaster muscles of WT or Cx3cr1‐GFP mice by cauterizing the primary vessels perfusing the tissue. (A) At 1 day post‐surgery the cremasteric vasculature was labelled fluorescently with anti‐Pecam‐1 antibody (i.s.), and fluorescent microspheres were administered (i.v.) 10 min before tissue collection. (B) Pimonidazole was used to visualise hypoxia in sham and PI tissues 1 day post‐surgery. (C) Example images and quantification of rounded and elongated Cx3cr1‐GFP^pos^ cells in 1 or 7 day PI tissues. (D) Example images of Pecam‐1 and ICAM‐1 labelled post‐capillary venules in sham 7 days PI tissues stimulated with LPS (300ng i.s.). Isosurfaces were built on the Pecam‐1 labelling and the intensity of Icam‐1 signal within this surface was quantified. (E) Lectin‐TRITC was given i.v. to label perfused vessels, and tissues were also labelled with anti‐Pecam‐1 antibody to show all vessels. Images were analysed along linear transects and co‐localisation of lectin‐TRITC and Pecam‐1 peaks recorded as perfused vessels with a functional lumen, and Pecam‐1 only peaks as non‐perfused vessels. (F) Representative images of vasculature in sham and PI tissues. (G) Example image of the spatial distribution of Cx3cr1‐GFP^pos^ cells and neutrophils in LPS stimulated 7 days PI tissue.

**Figure S2. Gating strategies for flow cytometry.** (A) In all flow cytometry analysis or sorting experiments (except isolation of cells for cell transfer in Figure 5) live leukocytes from lysed blood or digested tissues were identified by gating on FSC/SSC > CD45^pos^/DAPI^neg^ before further analysis as required. (i) The phenotype of tissue resident monocytes/macrophages in sham and PI cremasters or hind‐limb muscles was analysed by labelling with anti‐Gr1‐PE (clone RB6‐8C5), anti‐F4/80‐PE‐Cy7 and Cx3cr1‐GFP expression (Fig 1 and 4). (ii) Fluorescent microspheres (MSP) are delivered intravenously and surgical induction of ischaemia was carried out 48 h post i.v. MSP. The frequency of MSP labelling of Cx3cr1‐GFP^pos^/Gr1^high/low^ cells in the blood at the time of surgery and at 7D PI and in 7D PI tissues was analysed (Fig 1). (iii) Different macrophage subsets (MDCs and Cx3cr1‐GFP^neg^/TRITC^pos^ perivascular cells) for analysis or sorting were purified by gating on FSC/SSC > CD45^pos^/DAPI^neg^ > Cx3cr1‐GFP or dex‐TRITC (Fig 1 and 3). (iv) T‐cells and NK cells in the blood or 7D PI tissue were identified by CD3 and CD335 expression respectively. Data shows the percentage of all CD45^pos^ leukocytes which expressed Cx3cr1‐GFP, CD3 or CD335 in blood and 7D PI tissue.

**Figure S3. Cell transfer model.** (A) Cx3cr1‐GFP^pos^ cells for cell transfer were sorted based on rigorous FSC/SSC gating to exclude debris and cellular aggregates and select Cx3cr1‐GFP^pos^ cells as compared to WT GFP negative control tissues. No DAPI or antibody labels were used in order to limit potential functional effects post transfer. (B) 5x10^4^ cells purified from 7 days PI cremasters, or saline, were injected into the anterior tibialis muscle of Cx3cr1‐GFP^pos^ mice and left for 16 h before ischaemia (60 min) and reperfusion (120 min) was induced in the hind limb of the recipient mice by double ligation of the femoral artery. Tissue collection, weighing, enzymatic digestion and labelling of the cell suspension with DAPI, and fluorescent antibodies against CD45 and Ly6G (neutrophil specific clone 1A8) were used to analyse cell populations.

**Table S1. Intensity data for chemokine/cytokine immunoblot array of LPS‐stimulated cremasters.** Chronic ischaemia, or sham surgery, was induced in the cremasters of WT mice. After 7 days, circulating neutrophils were depleted with anti‐Ly6G antibody (100 µg i.p. 24 h) and some tissues were monocyte/macrophage‐depleted with locally applied clodronate liposomes. Cremasters were subsequently stimulated with LPS (300ng i.s., 4 h) or saline. Tissues were collected, homogenised and analysed using a chemokine/cytokine array immunoblot according to manufacturer's instructions (R&D Systems). The mean intensity values for each chemokine/cytokine in each treatment group were normalised to total protein and the intensity of the control spots per blot. Each blot contained pooled tissue from two animals and was repeated twice.

**Table S2. Intensity data for chemokine/cytokine immunoblot array from acute ischaemia/reperfusion‐stimulated hind limbs.** Cx3cr1‐GFP^pos^ cells were purified from 7 days PI cremasters by FACS. 5x10^4^ cells, or saline, were injected into the tibialis anterior muscle of Cx3cr1‐GFP or WT mice and left overnight. Acute ischaemia (60 min) and reperfusion (120 min) was induced buy double ligation of the femoral artery and vein. Muscles were collected, homogenised and analysed using a chemokine/cytokine array immunoblot according to manufacturer's instructions (R&D Systems). The intensity values for each chemokine/cytokine in each treatment group were normalised to total protein and the intensity of the control spots per blot. Each blot contained pooled tissues from 6 animals per group.

**Video S1. Neutrophil extravasation in sham tissues.** Chronic ischaemia or sham surgery was induced in the cremasters of LysM‐GFP mice, which have GFP positive neutrophils. After 7 days the tissues were labelled with anti‐Pecam‐1 antibody, exteriorized for viewing and acutely stimulated with topical LTB_4_ (100 nM). Tissues were imaged at 3 min intervals. The video shows neutrophil extravasation in a sham tissue. A still from this video is shown in Figure 2C.

**Video S2. Neutrophil extravasation in PI tissues.** Chronic ischaemia or sham surgery was induced in the cremasters of LysM‐GFP mice, which have GFP positive neutrophils. After 7 days the tissues were labelled with anti‐Pecam‐1 antibody, exteriorized for viewing and acutely stimulated with topical LTB_4_ (100 nM). Tissues were imaged at 3 min intervals. Video shows neutrophil extravasation in a 7 days PI tissue. A still from this video is shown in Figure 2C.

## References

[1] Nourshargh S , Hordijk PL , Sixt M . Breaching multiple barriers: leukocyte motility through venular walls and the interstitium. Nat Rev Mol Cell Biol 2010; 11 **:** 366–378. 20414258 10.1038/nrm2889

[2] Nourshargh S , Alon R . Leukocyte migration into inflamed tissues. Immunity 2014; 41 **:** 694–707. 25517612 10.1016/j.immuni.2014.10.008

[3] Mantovani A , Allavena P , Sica A , et al. Cancer‐related inflammation. Nature 2008; 454 **:** 436–444. 18650914 10.1038/nature07205

[4] Hauser SL , Oksenberg JR . The neurobiology of multiple sclerosis: genes, inflammation, and neurodegeneration. Neuron 2006; 52 **:** 61–76. 17015227 10.1016/j.neuron.2006.09.011

[5] Szekanecz Z , Besenyei T , Szentpetery A , et al. Angiogenesis and vasculogenesis in rheumatoid arthritis. Curr Opin Rheumatol 2010; 22 **:** 299–306. 20305562 10.1097/BOR.0b013e328337c95a

[6] Hansson GK . Inflammation, atherosclerosis, and coronary artery disease. N Engl J Med 2005; 352 **:** 1685–1695. 15843671 10.1056/NEJMra043430

[7] Vinten‐Johansen J , Jiang R , Reeves JG , et al. Inflammation, proinflammatory mediators and myocardial ischaemia–reperfusion Injury. Hematol Oncol Clin North Am 2007; 21 **:** 123–145. 17258123 10.1016/j.hoc.2006.11.010

[8] Berger JS , Hiatt WR . Medical therapy in peripheral artery disease. Circulation 2012; 126 **:** 491–500. 22825411 10.1161/CIRCULATIONAHA.111.033886

[9] Frantz S , Nahrendorf M . Cardiac macrophages and their role in ischaemic heart disease. Cardiovasc Res 2014; 102 **:** 240–248. 24501331 10.1093/cvr/cvu025 PMC3989449

[10] Patel AS , Smith A , Nucera S , et al. TIE2‐expressing monocytes/macrophages regulate revascularization of the ischemic limb. EMBO Mol Med 2013; 5 **:** 858–869. 23653322 10.1002/emmm.201302752 PMC3779448

[11] Silvestre JS , Smadja DM , Levy BI . Postischemic revascularization: from cellular and molecular mechanisms to clinical applications. Physiol Rev 2013; 93 **:** 1743–1802. 24137021 10.1152/physrev.00006.2013

[12] Jackson JR , Seed MP , Kircher CH , et al. The codependence of angiogenesis and chronic inflammation. FASEB J 1997; 11 **:** 457–465. 9194526

[13] Naldini A , Carraro F . Role of inflammatory mediators in angiogenesis. Curr Drug Targets Inflamm Allergy 2005; 4 **:** 3–8. 15720228 10.2174/1568010053622830

[14] Abtin A , Jain R , Mitchell AJ , et al. Perivascular macrophages mediate neutrophil recruitment during bacterial skin infection. Nat Immunol 2014; 15 **:** 45–53. 24270515 10.1038/ni.2769 PMC4097073

[15] Chen GY , Nunez G . Sterile inflammation: sensing and reacting to damage. Nat Rev Immunol 2010; 10 **:** 826–837. 21088683 10.1038/nri2873 PMC3114424

[16] Woodfin A , Voisin MB , Beyrau M , et al. The junctional adhesion molecule JAM‐C regulates polarized transendothelial migration of neutrophils in vivo. Nat Immunol 2011; 12 **:** 761–769. 21706006 10.1038/ni.2062 PMC3145149

[17] Leoni G , Patel HB , Sampaio AL , et al. Inflamed phenotype of the mesenteric microcirculation of melanocortin type 3 receptor‐null mice after ischaemia–reperfusion. FASEB J 2008; 22 **:** 4228–4238. 18757499 10.1096/fj.08-113886 PMC2700033

[18] Jung S , Aliberti J , Graemmel P , et al. Analysis of fractalkine receptor CX(3)CR1 function by targeted deletion and green fluorescent protein reporter gene insertion. Mol Cell Biol 2000; 20 **:** 4106–4114. 10805752 10.1128/mcb.20.11.4106-4114.2000 PMC85780

[19] Faust N , Varas F , Kelly LM , et al. Insertion of enhanced green fluorescent protein into the lysozyme gene creates mice with green fluorescent granulocytes and macrophages. Blood 2000; 96 **:** 719–726. 10887140

[20] Lotfi S , Patel AS , Mattock K , et al. Towards a more relevant hind limb model of muscle ischaemia. Atherosclerosis 2012; 227 **:** 1–8. 23177969 10.1016/j.atherosclerosis.2012.10.060

[21] Hellingman AA , Bastiaansen AJ , de Vries MR , et al. Variations in surgical procedures for hind limb ischaemia mouse models result in differences in collateral formation. Eur J Vasc Endovasc Surg 2010; 40 **:** 796–803. 20705493 10.1016/j.ejvs.2010.07.009

[22] Tacke F , Alvarez D , Kaplan TJ , et al. Monocyte subsets differentially employ CCR2, CCR5, and Cx3cr1 to accumulate within atherosclerotic plaques. J Clin Invest 2007; 117 **:** 185–194. 17200718 10.1172/JCI28549 PMC1716202

[23] Tacke F , Ginhoux F , Jakubzick C , et al. Immature monocytes acquire antigens from other cells in the bone marrow and present them to T cells after maturing in the periphery. J Exp Med 2006; 203 **:** 583–597. 16492803 10.1084/jem.20052119 PMC2118235

[24] Arnold L , Henry A , Poron F , et al. Inflammatory monocytes recruited after skeletal muscle injury switch into antiinflammatory macrophages to support myogenesis. J Exp Med 2007; 204 **:** 1057–1069. 17485518 10.1084/jem.20070075 PMC2118577

[25] Bottcher JP , Beyer M , Meissner F , et al. Functional classification of memory CD8(+) T cells by Cx3cr1 expression. Nat Commun 2015; doi: 10.1038/ncomms9306. 10.1038/ncomms9306 PMC4667439 26404698

[26] Cochain C , Rodero MP , Vilar J , et al. Regulation of monocyte subset systemic levels by distinct chemokine receptors controls post‐ischaemic neovascularization. Cardiovasc Res 2010; 88 **:** 186–195. 20501509 10.1093/cvr/cvq153

[27] Nahrendorf M , Swirski FK , Aikawa E , et al. The healing myocardium sequentially mobilizes two monocyte subsets with divergent and complementary functions. J Exp Med 2007; 204 **:** 3037–3047. 18025128 10.1084/jem.20070885 PMC2118517

[28] Livak KJ , Schmittgen TD . Analysis of relative gene expression data using real‐time quantitative PCR and the 2(‐Delta Delta C(T)) Method. Methods 2001; 25 **:** 402–408. 11846609 10.1006/meth.2001.1262

[29] Hiatt WR , Armstrong EJ , Larson CJ , et al. Pathogenesis of the limb manifestations and exercise limitations in peripheral artery disease. Circ Res 2015; 116 **:** 1527–1539. 25908726 10.1161/CIRCRESAHA.116.303566

[30] Capoccia BJ , Gregory AD , Link DC . Recruitment of the inflammatory subset of monocytes to sites of ischaemia induces angiogenesis in a monocyte chemoattractant protein‐1‐dependent fashion. J Leukoc Biol 2008; 84 **:** 760–768. 18550788 10.1189/jlb.1107756 PMC2516891

[31] Rademakers T , Douma K , Hackeng TM , et al. Plaque‐associated vasa vasorum in aged apolipoprotein E‐deficient mice exhibit proatherogenic functional features in vivo. Arterioscler Thromb Vasc Biol 2013; 33 **:** 249–256. 23241413 10.1161/ATVBAHA.112.300087

[32] Hogan RD , Hirschmann L . Arteriolar proliferation in the rat cremaster muscle as a long‐term autoregulatory response to reduced perfusion. Microvasc Res 1984; 27 **:** 290–296. 6203016 10.1016/0026-2862(84)90061-x

[33] Schaper W. Collateral circulation: past and present. Basic Res Cardiol 2009; 104 **:** 5–21. 19101749 10.1007/s00395-008-0760-x PMC2755790

[34] Segel GB , Halterman MW , Lichtman MA . The paradox of the neutrophil's role in tissue injury. J Leukoc Biol 2011; 89 **:** 359–372. 21097697 10.1189/jlb.0910538 PMC6608002

[35] Gong Y , Koh DR . Neutrophils promote inflammatory angiogenesis via release of preformed VEGF in an in vivo corneal model. Cell Tissue Res 2010; 339 **:** 437–448. 20012648 10.1007/s00441-009-0908-5

[36] Christoffersson G , Vagesjo E , Vandooren J , et al. VEGF‐A recruits a proangiogenic MMP‐9‐delivering neutrophil subset that induces angiogenesis in transplanted hypoxic tissue. Blood 2012; 120 **:** 4653–4662. 22966168 10.1182/blood-2012-04-421040 PMC3512240

[37] Baluk P , Phillips K , Yao LC , et al. Neutrophil dependence of vascular remodeling after Mycoplasma infection of mouse airways. Am J Pathol 2014; 184 **:** 1877–1889. 24726646 10.1016/j.ajpath.2014.02.010 PMC4044721

[38] Boyle JJ , Wilson B , Bicknell R , et al. Expression of angiogenic factor thymidine phosphorylase and angiogenesis in human atherosclerosis. J Pathol 2000; 192 **:** 234–242. 11004701 10.1002/1096-9896(2000)9999:9999<::AID-PATH699>3.0.CO;2-9

[39] O'Brien KD , McDonald TO , Chait A , et al. Neovascular expression of E‐selectin, intercellular adhesion molecule‐1, and vascular cell adhesion molecule‐1 in human atherosclerosis and their relation to intimal leukocyte content. Circulation 1996; 93 **:** 672–682. 8640995 10.1161/01.cir.93.4.672

[40] Inoue M , Ishida T , Yasuda T , et al. Endothelial cell‐selective adhesion molecule modulates atherosclerosis through plaque angiogenesis and monocyte–endothelial interaction. Microvasc Res 2010; 80 **:** 179–187. 20406651 10.1016/j.mvr.2010.04.005

[41] Bobryshev YV , Cherian SM , Inder SJ , et al. Neovascular expression of VE‐cadherin in human atherosclerotic arteries and its relation to intimal inflammation. Cardiovasc Res 1999; 43 **:** 1003–1017. 10615428 10.1016/s0008-6363(99)00125-x

[42] Sadat U , Jaffer FA , van Zandvoort MA , et al. Inflammation and neovascularization intertwined in atherosclerosis: imaging of structural and molecular imaging targets. Circulation 2014; 130 **:** 786–794. 25156914 10.1161/CIRCULATIONAHA.114.010369 PMC4212981

[43] Sumagin R , Sarelius IH . Tnf‐alpha activation of arterioles and venules alters distribution and levels of Icam‐1 and affects leukocyte–endothelial cell interactions. Am J Physiol Heart Circ Physiol 2006; 291 **:** H2116–H2125. 16766643 10.1152/ajpheart.00248.2006 PMC1593218

[44] Woodfin A , Voisin M‐B , Engelhardt B , et al. Endothelial cell activation leads to neutrophil transmigration as supported by the sequential roles of ICAM‐2, JAM‐A and Pecam‐1. Blood 2009; 113 **:** 6246–6257. 19211506 10.1182/blood-2008-11-188375 PMC2699241

[45] Sadik CD , Kim ND , Luster AD . Neutrophils cascading their way to inflammation. Trends Immunol 2011; 32 **:** 452–460. 21839682 10.1016/j.it.2011.06.008 PMC3470857

[46] Helisch A , Wagner S , Khan N , et al. Impact of mouse strain differences in innate hindlimb collateral vasculature. Arterioscler Thromb Vasc Biol 2006; 26 **:** 520–526. 16397137 10.1161/01.ATV.0000202677.55012.a0

[47] Scholz D , Ziegelhoeffer T , Helisch A , et al. Contribution of arteriogenesis and angiogenesis to postocclusive hindlimb perfusion in mice. J Mol Cell Cardiol 2002; 34 **:** 775–787. 12099717 10.1006/jmcc.2002.2013

[48] Troidl K , Schaper W . Arteriogenesis versus angiogenesis in peripheral artery disease. Diabetes Metab Res Rev 2012; 28 **:** 27–29. 22271719 10.1002/dmrr.2232

