# Supplementary material for: Underlying chronic inflammation alters the profile and mechanisms of acute neutrophil recruitment
Source: J Pathol. 2016 Oct 19;240(3):291–303. doi: 10.1002/path.4776 (PMC5082550; doi:10.1002/path.4776)
Supplement: Supplementary file 1 — Supplementary materials and methods [file PATH-240-291-s001.docx]

**Supplementary information**

# Detailed methods

**Reagents**

Il-1b, LPS, LTB_4_, Tnf, DAPI, collagenase, and DNAse were purchased from Sigma-Aldrich. Fluorescent beads, isolectin-TRITC, dextran-TRITC and CountBright flow counting beads were purchased from Molecular Probes. Clodronate liposomes were purchased from Encapsular Nanosciences (Nashville, Tenessee, United States). Chemokine/cytokine array kits were purchased from R&D Systems (Abingdon, Oxfordshire, United Kingdom). Pimonidazole was purchased from Hypoxyprobe (Burlington, Massachusetts, United States). Halt^TM^ protease and phosphatase inhibitor was purchased from Thermo Scientific (Paisley, Renfrewshire, United Kingdom). RNeasy miniprep kit and iQ-SYBR green were purchased from BioRad (Hemel Hempstead, Hertfordshire, United Kingdom). Fluoresbrite microspheres (0.5 µm) to selectively label Gr1^low^ monocytes in the circulation were purchased from Polysciences (Hirschberg an der Bergstrasse, Germany).

Antibodies were purchased from eBioscience (Hatfield, Hertfordshire, United Kingdom): anti-Pecam-1 (clone C390, IgG2A, catalogue 16-0311), anti-TNF (clone MP6-XT22, IgG1, catalogue 16-7321). Biolegend (London, United Kingdom): anti-CD45 (clone 30-F11, catalogue 103112), anti-Ly6G (clone 1A8, catalogue 127608), anti-Icam-1 (clone YN1/1.7.4, catalogue 116112), anti-Gr1 (clone RB6-8C5, catalogue 108408), anti-F4-80 (clone BM8, catalogue 123114), anti-CD3 (clone 17A2, catalogue 100206) and anti-CD335 (clone 29A1.4, catalogue 137604). Rat IgG1 (clone RTK2071 , catalogue 400414), IgG2a (clone RTK2758, catalogue 400508) and IgG2b isotype controls (clone RTK2071, catalogue 400402).Abcam (Cambridge, Cambridgeshire, United Kingdom): anti-laminin (polyclonal, catalogue ab30320). Pharmingen/BD Biosciences (Oxford, Oxfordshire, United Kingdom): anti-CD16/CD32 FC-Block (catalogue 553142). R&D Systems (Abingdon, Oxfordshire, United Kingdom): anti-CCL5 (clone 53405, catalogue Mab478), anti-CXCL2 (clone 40605, catalogue Mab452l), anti-CCL3 (clone 39624, catalogueMab450). Or were provided by Research Cell Services, Cancer Research UK London Research Institute: anti-S100a9 (clone 2B10). Primary antibodies were either commercially conjugated or labelled in house with Molecular Probes AlexaFluor antibody conjugation kits.

**Animals**

Male WT C57BL/6 mice were purchased from Charles River Laboratories (stock ID 027). Heterozygous mice in which the gene for GFP has been knocked into the fractalkine receptor (Cx3cr1) locus (*Cx3cr1-GFP*), resulting in GFP expression in circulating monocytes and monocyte derived cells in the tissue were used (18), and were obtained from the European Mutant Mouse Archive (EMMA, Orleans, France). Heterozygous mice in which the gene for GFP has been knocked-in to the lysozyme M (*LysM*) locus (*LysM-GFP*) (19), resulting in the exhibition of GFP in myelomonocytic cells, with high levels of GFP in neutrophils and to a lesser extent monocytes, were a gift from Professor Markus Sperandio. Differential levels of GFP expression can be used to distinguish neutrophils and monocytes in these animals (16). All genetically modified animals were on a C57BL/6 background. All animals were housed in individually ventilated cages and facilities were regularly monitored for health status and infections. All experiments were performed under the UK legislation for the protection of animals, and at the end of all *in vivo* procedures animals were humanely killed by cervical dislocation in accordance with UK Home Office regulations.

**Induction of chronic ischaemia and angiogenesis**

Ischaemia and angiogenesis were induced in the mouse cremaster. Animals were anesthetized in an Isoflurane induction chamber then placed ventral side up on a sterile surgical field. Anaesthesia was maintained with Isoflurane. The scrotum and surrounding area were shaved with an electric clipper, and sprayed with ethanol before making an incision in the scrotum. The testes were separated from surrounding connective tissue to enable them to descend through the incision, and lifted upwards to expose the main vessels supplying the cremaster which run along the dorsal side of the tissue. A hyfrecator (Conmed) was used to cauterize a single point on the main arteriole and venule supplying the tissue as proximal to the body as possible. Due to the close proximity and small scale of the arteriole and vein (~ 300µm combined diameter), it was not possible to cauterize or ligate the arteriole in isolation from the vein. In sham surgery the ventral side of the tissue, where no major vessels are affected, was cauterized. Following cauterization, the testes were returned to the body cavity and the incision was closed with 3 or 4 sutures of Prolene and Vetbond skin adhesive. At the time of tissue analysis visual confirmation was made that occlusion of the main vessels had persisted. In some cases, circulating monocytes were depleted prior to surgery using 100 µl Clodronate liposomes given i.v. 24 h prior to surgery, immediately after surgery, and at 48 h time points for the following 7 days. In some experiments Clodronate liposomes were given i.v. 40 h after surgery and at 48 h time points for the following 7 days.

Chronic ischaemia and angiogenesis were induced in the mouse hind limb by occlusion and excision of part of the femoral artery and vein (20, 21). Briefly the animal is anaesthetized as above, and an area on the inside of the upper hind limb shaved. A small incision is made and the muscles moved to the side using retractors to expose the femoral artery and vein. The vessels were ligated proximal to the inguinal ligament and again approximately 5 mm more distal. The vessel section within is excised and the cut ends cauterized. The wound was closed with Prolene sutures and Vetbond as above. Sham animals underwent exposure of the femoral vessels exposed but no ligation or excision.

**Induction of acute inflammatory responses**

Animals were anaesthetized with ketamine and xylazine (i.m) or inhaled isofluorane. Cremasteric inflammation was induced by intrascrotal (i.s.) injection of IL-1β (50 ng), TNF (300 ng), or LPS (300 ng) in 300 µl saline. In live imaging experiments LTB_4_ (100 nM) was applied topically to the cremaster during imaging. In some studies, neutralizing antibodies against CCL3 (20 µg), CCL5 (20 µg), CXCL2 (10 µg) and TNF (10 µg) were co-applied with 300 ng LPS. Hind-limb muscles were stimulated with 100 µg LPS in 30 µl saline (i.m.) for 4 hours before collection/analysis. Of note these studies were primarily carried out using contralateral cremasters and legs within the same animal as the sham and PI tissues, so circulating neutrophil numbers were equivalent.

**Immunofluorescence confocal microscopy**

Immunofluorescent confocal microscopy was employed to analyse the extent and characteristics of vascularization and the frequency, distribution and morphology of different leukocyte subsets in sham or PI tissues. The cremasteric vasculature was visualized by local injection (i.s.) of fluorescently conjugated anti-Pecam-1 antibody (2 µg in 300 µl, 2 h), or *ex vivo* labelling (5 µg/ml), or by administration of isolectin-TRITC (100 µg, i.v.) 1 h before exteriorization and imaging. Intrascrotal delivery of anti-Pecam-1 antibody labels capillaries and venules strongly, but labels arterioles less strongly, despite their high level expression of this protein. We believe that this is a result of more limited access of exogenously applied antibody to the endothelial cells due to more complete coverage with smooth muscle cells.

Vessel perfusion was assessed by i.v. injection of fluorescent microbeads 10 min before exteriorization and imaging. Tissue hypoxia was visualized with the fluorescent probe pimonidazole. Pimonidazole was delivered locally before the animals were killed (500 µg i.s., 15 min) and tissues rapidly collected and fixed with PFA (4 %). Tissues were blocked and permeabilised with FCS (20 %) and Triton-X-100 (0.5 %), and incubated with fluorescent anti-pimonidazole antibody (10 µg/ml, overnight).

Monocyte derived cells in the tissue were visualized by GFP expression in Cx3cr1-GFP animals. Phagocytic cells were labelled with dextran-TRITC (20 µg i.s.) for 2 h. Neutrophils were visualized by GFP expression in LysM-GFP animals, or by *ex vivo* labelling with antibodies against the neutrophil marker S100a9 (1 µg/ml). LysM-GFP animals were used whenever it was unnecessary to distinguish between neutrophils and monocytes to remove the need for additional labelling steps. S100a9 antibody labelling was used for neutrophil detection in tissue histology studies as we find it is significantly more effective than clone 1A8 in this protocol, and this antibody was specific to neutrophils. Fixation, blocking and permeabilisation were carried out as above.

The vasculature and leukocyte populations of the hind-limb muscles were analyzed by fixation in methanol for 20 min to visualize the capillaries and muscle fibers, or in PFA (4 %) overnight to visualize neutrophils or Cx3cr1-GFP^pos^ cells. After fixation the muscles were embedded in OCT and frozen in liquid nitrogen. Tissues were cut into 30-100 µm sections using a LEICA CM 1510S Cryotome, and mounted on Poly-L-lysine slides. Sections were labelled with directly conjugated antibodies against S100a9 (5 µg/ml), Pecam-1 (10 µg/ml) or laminin (5 µg/ml). GFP signal was used to identify Cx3cr1-GFP^pos^ or LysM-GFP^pos^ cells.

At least four images of each tissue or muscle section were collected from at least 3 mice per group using a Leica SP5 confocal microscope incorporating a 20× water-dipping objective (NA 1.0). In live imaging experiments cremasters were exteriorized and images were captured at 3 min intervals for up to 2 h of LTB_4_ stimulation as previously described (16). Quantification of images was carried out using Imaris (Bitplane) 3D analysis software or Leica LASF-Lite software. The frequency of a particular cell type, vessel frequency or diameter per image was analysed as described in each figure legend.

**Flow Cytometry**

In studies of cremasteric or hind-limb leukocyte populations tissues were finely chopped, digested with 200 U/ml each of Collagenase and DNAse for 30 min at 37°C, then passed through a 40 µm sieve. In some studies, phagocytic cells were labelled with dextran-TRITC (40,000 MW, 20 µg i.s.) for 2 hours prior to tissue collection. Cell suspensions were blocked with anti-CD16/CD32 FC block (10 µg/ml) and labelled with DAPI and fluorescent antibodies against CD45, clone RB6-8C5 which detects Gr1 on monocytes and neutrophils, clone 1A8 that detects neutrophils only, F4/80 or the appropriate isotype controls, all at 10 µg/ml. Leukocytes were identified by FSC and SSC characteristics and CD45-Alexa700 or CD45-APC positive staining (633/700 or 633/670nm excitation/emission), dead cells were excluded by DAPI staining (407/450 excitation/emission), Cx3cr1-GFP cells were identified by GFP expression (488-530 nm excitation/emission), phagocytic cells were identified by TRITC signal (560/585 nm excitation/emission). Gr1-PE (560/585 nm excitation/emission) and F4-80-PeCy7 (488/780 nm excitation/emission) were quantified on each subset of interest as compared to an isotype control. T-cells were identified by CD3-PE expression and NK cells were identified by CD335-PE expression (560/585 nm excitation/emission). Samples were analyzed using a BD LSR-Fortessa, or purified using a BD FACS-Aria (BD Biosciences). Data was analyzed using FloJo analysis software (Treestar).

**Specific labelling and tracking of Gr1^low^ monocytes**

Gr1^low^ monocytes in the circulation were selectively labelled with an i.v. injection of 20 µl fluorescent microspheres (MSP, 0.5 µm diameter) in 200 µl saline. This labelling technique does not affect transmigration (22, 23, 24). Surgical induction of ischaemia is carried out at 48h post injection. The frequency of Cx3cr1-GFP^pos^/Gr1^low^/MSP^pos^ cells in the blood at the time of surgery and at 7 days PI was compared to the frequency in 7 days PI tissues in order to determine if Gr1^low^ monocytes were recruited to the tissues.

**Cell transfer**

Cremasters from Cx3cr1-GFP mice were digested at 7 days PI as described above, and GFP^pos^ cells were purified using a FACS-Aria. No other antibodies or markers were used in order to preserve normal cell function. 5x10^4^ Cx3cr1-GFP^pos^ cells in 50 µl saline were injected into the anterior tibialis muscle of naïve Cx3cr1-GFP^pos^ mice across 3 injection sites and left for 16 h.

Acute ischaemia and reperfusion of a hind limb was induced by exposure of the femoral artery and vein, double ligation proximal to the inguinal ligament and approximately 5 mm distal with suture silk, and maintained for 60 min. Ligations were removed allowing reperfusion of the limb for 120 min before collection of the anterior tibialis muscle. Sham animals underwent exposure of the femoral vessels but no ligation. The muscle was collected, weighed, and enzymatically digested as above. Cell suspensions were blocked with anti-CD16/CD32 FC block (10 µg/ml) and labelled with DAPI and fluorescent antibodies against CD45-APC, and Ly6G-PE (10 µg/ml). Flow counting beads (10,000 beads in 300 µl cell suspension) were used to quantify the number of neutrophils, Cx3cr1-GFP^pos^ and other CD45^pos^ leukocytes present per mg of tissue. Undigested muscle samples were retained at -80°C for cytokine/chemokine array analysis.

**Cytokine/chemokine array analysis**

7 days after induction of ischaemia circulating neutrophils were depleted with an anti-Ly6G antibody (100 µg i.p.). In some cases, cremasteric monocytes/macrophages were depleted by Clodronate liposomes (50 µl i.s. and 100 µl i.v. on day 6, and 50 µl i.s. on days 7 and 8 PI). On day 8 cremasters were stimulated with LPS (300 ng) or saline for 4 h before tissue collection. In other experiments muscle samples from of sham or acute-IR stimulated hind-limb muscles were analysed. Tissues were chopped finely and homogenized in 500 µl PBS and Halt^TM^ protease inhibitor, using a Percellys-24 homogenizer (Bertin Technologies). Samples were centrifuged at 10,000 rcf for 10 min. A BCA protein assay was carried out for each sample and the intensity of each signal was normalised to the amount of protein added and the control spots on each array. Samples were analysed using an R&D mouse chemokine/cytokine array according to manufacturer’s instructions. Blots were analysed using ImageJ.

**RT-qPCR**

Sham or PI cremasters from Cx3cr1-GFP mice were labelled with dex-TRITC (20 µg i.s.), and stimulated with LPS or saline (300 ng, 4 h). Tissues were collected, digested and labelled with DAPI and anti-CD45 as above. CD45^pos^/DAPI^neg^/Cx3cr1-GFP^pos^ and CD45^pos^/DAPI^neg^/dex-TRITC^pos^/Cx3cr1-GFP^neg^ cells were purified using a BD FACS-Aria (BD Biosciences). In some experiments Cx3cr1-GFP^pos^/Gr1^low^ cells were differentially purified from unstimulated or LPS stimulated PI cremasters. Cell purity was >80 %. Purified cells were lysed in RPE buffer and RNA was extracted using an RNeasy miniprep kit (Qiagen) following the manufacturer's instructions, and reverse-transcribed using i-Script cDNA synthesis kit (Biorad). Quantitative PCR was performed using the primers listed below in conjunction with iQ-SYBR green supermix (Biorad) on an ABI 7900HT qPCR machine (Applied Biosystems) with cycling conditions as follows: 2 min, 50°C, 10 min, 95°C denaturation and then 50 repeats of a two-step amplification cycle (95°C for 15 s and 60°C for 60 s). Gene expression levels were normalized against the reference gene GAPDH, and results are shown as the fold increase in expression compared to unstimulated cells using the 2^-∆∆CT^ method (28). The following primers were used: *Gapdh* forwards: 5’-TCG TGG ATC TGA CGT GCC GCC TG-3’ reverse: 5’-CAC CAC CCT GTT GCT GTA GCC GTA T-3’. IL-1β forwards: 5’-GTA ATG AAA GAC GGC ACA CC-3’ reverse: 5’-CTC TCG TTG TGA GGT GCT G-3’. CCL3 forwards: 5’- CAT ATG GAG CTG ACA CCC CG-3’ reverse: 5’-CGT GGA ATC TTC CGG CTG TA-3’. CCL5 forwards: 5’-TGC CCA CGT CAA GGA GTA TTT C-3’ reverse: 5’-AAC CCA CTT CTT CTC TGG GTT G-3’. CXCL1 forwards: 5’-TGT CAG TGC CTG CAG ACC AT-3’ reverse: 5’-CCT GAG GGC AAC ACC TTC A-3’. CXCL2 forwards: 5’-CCC TCA ACG GAA GAA CCA AA-3’ reverse: 5’-AGG CAC ATC AGG TAC GAT CCA-3’. TNF forwards: 5’-GCC TCT TCT CAT TCC TGC TTG-3’ reverse: 5’-CTG ATG AGA GGG AGG CCA TT-3’.

**Statistical analysis**

Results are presented as mean ± s.e.m. Statistical significance was assessed using Student’s *t*-test or by one-way analysis of variance (ANOVA) with the Newman-Keuls multiple comparison test. P values below 0.05 were considered significant.
