# Supplementary figures and images for: Underlying chronic inflammation alters the profile and mechanisms of acute neutrophil recruitment
Source: J Pathol. 2016 Oct 19;240(3):291–303. doi: 10.1002/path.4776 (PMC5082550; doi:10.1002/path.4776)

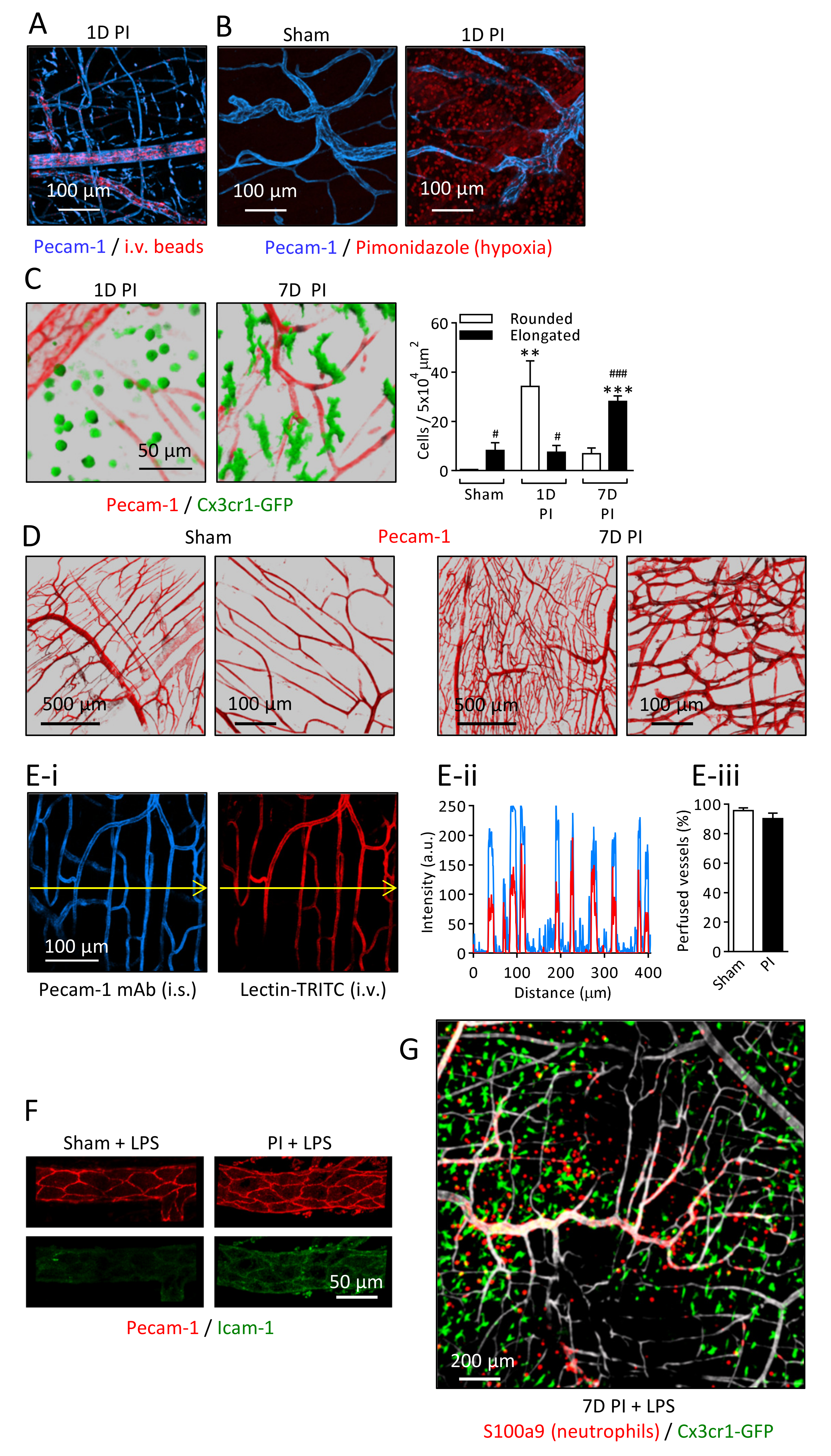

Supplement: Supplementary file 3 — Figure S1. Additional images and data. Chronic ischaemia was induced in cremaster muscles of WT or Cx3cr1‐GFP mice by cauterizing the primary vessels perfusing the tissue. (A) At 1 day post‐surgery the cremasteric vasculature was labelled fluorescently with anti‐Pecam‐1 antibody (i.s.), and fluorescent microspheres were administered (i.v.) 10 min before tissue collection. (B) Pimonidazole was used to visualise hypoxia in sham and PI tissues 1 day post‐surgery. (C) Example images and quantification of rounded and elongated Cx3cr1‐GFPpos cells in 1 or 7 day PI tissues. (D) Example images of Pecam‐1 and ICAM‐1 labelled post‐capillary venules in sham 7 days PI tissues stimulated with LPS (300ng i.s.). Isosurfaces were built on the Pecam‐1 labelling and the intensity of Icam‐1 signal within this surface was quantified. (E) Lectin‐TRITC was given i.v. to label perfused vessels, and tissues were also labelled with anti‐Pecam‐1 antibody to show all vessels. Images were analysed along linear transects and co‐localisation of lectin‐TRITC and Pecam‐1 peaks recorded as perfused vessels with a functional lumen, and Pecam‐1 only peaks as non‐perfused vessels. (F) Representative images of vasculature in sham and PI tissues. (G) Example image of the spatial distribution of Cx3cr1‐GFPpos cells and neutrophils in LPS stimulated 7 days PI tissue. [file PATH-240-291-s002.tif]

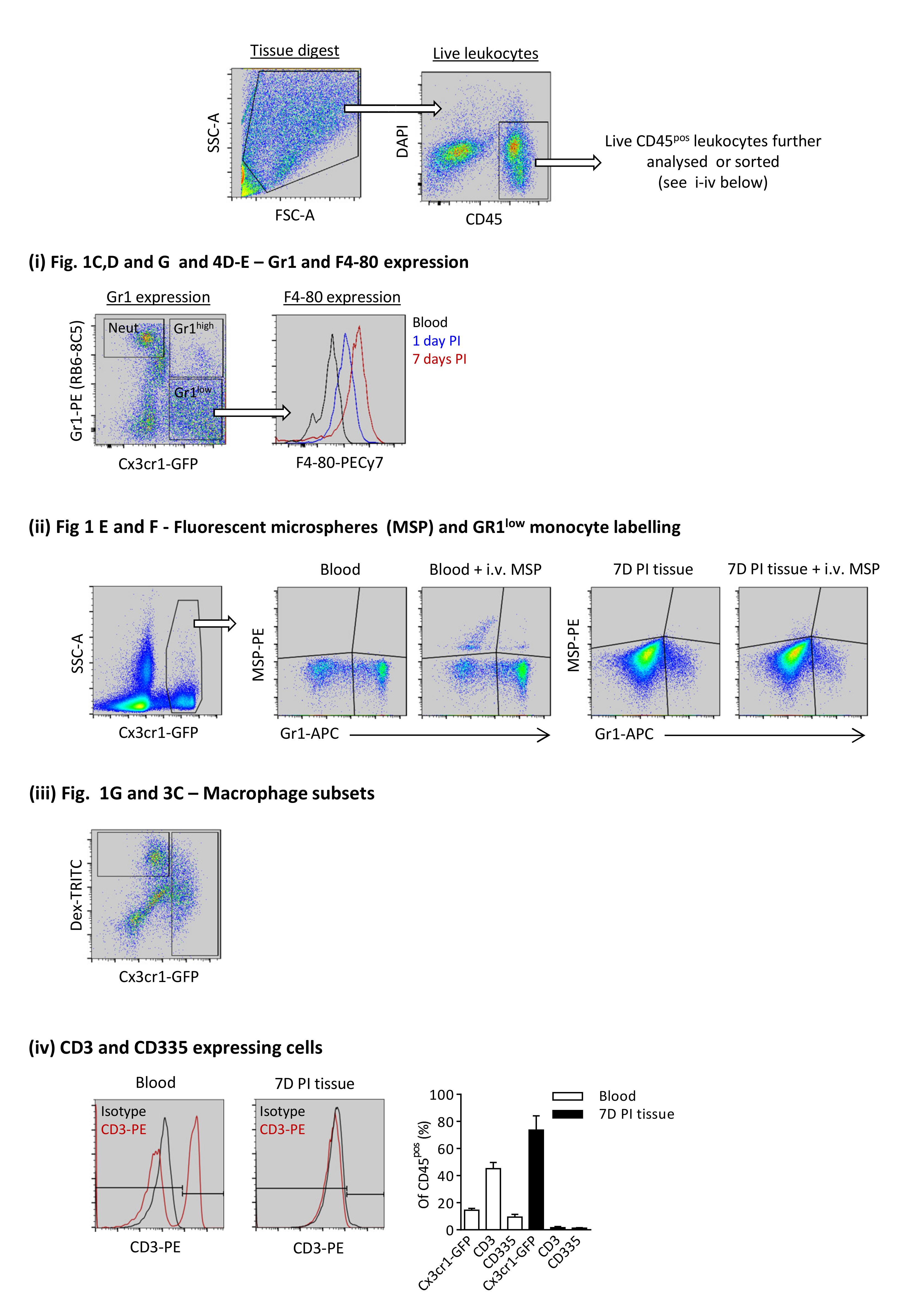

Supplement: Supplementary file 4 — Figure S2. Gating strategies for flow cytometry. (A) In all flow cytometry analysis or sorting experiments (except isolation of cells for cell transfer in Figure 5) live leukocytes from lysed blood or digested tissues were identified by gating on FSC/SSC > CD45pos/DAPIneg before further analysis as required. (i) The phenotype of tissue resident monocytes/macrophages in sham and PI cremasters or hind‐limb muscles was analysed by labelling with anti‐Gr1‐PE (clone RB6‐8C5), anti‐F4/80‐PE‐Cy7 and Cx3cr1‐GFP expression (Fig 1 and 4). (ii) Fluorescent microspheres (MSP) are delivered intravenously and surgical induction of ischaemia was carried out 48 h post i.v. MSP. The frequency of MSP labelling of Cx3cr1‐GFPpos/Gr1high/low cells in the blood at the time of surgery and at 7D PI and in 7D PI tissues was analysed (Fig 1). (iii) Different macrophage subsets (MDCs and Cx3cr1‐GFPneg/TRITCpos perivascular cells) for analysis or sorting were purified by gating on FSC/SSC > CD45pos/DAPIneg > Cx3cr1‐GFP or dex‐TRITC (Fig 1 and 3). (iv) T‐cells and NK cells in the blood or 7D PI tissue were identified by CD3 and CD335 expression respectively. Data shows the percentage of all CD45pos leukocytes which expressed Cx3cr1‐GFP, CD3 or CD335 in blood and 7D PI tissue. [file PATH-240-291-s009.tif]

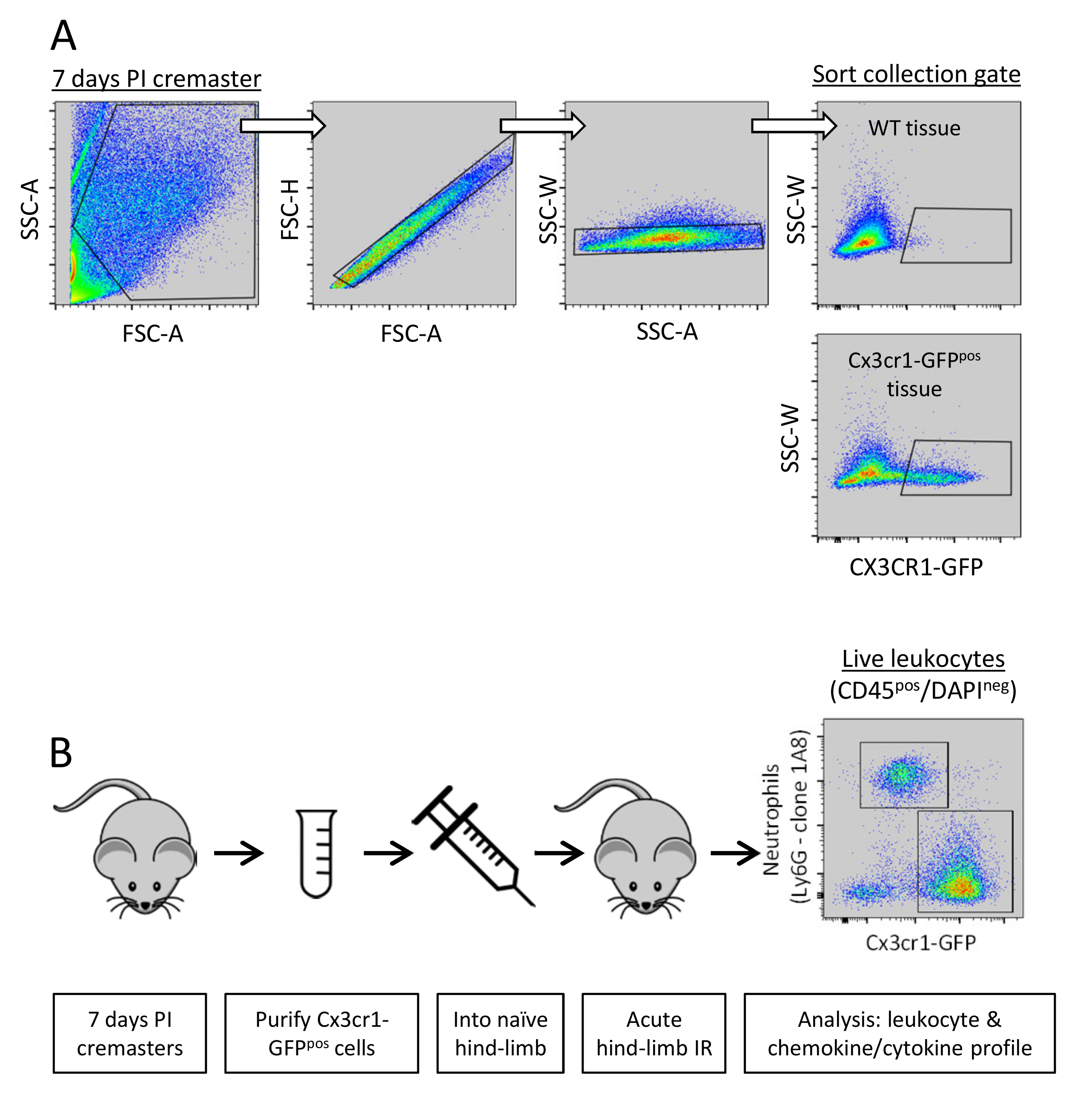

Supplement: Supplementary file 5 — Figure S3. Cell transfer model. (A) Cx3cr1‐GFPpos cells for cell transfer were sorted based on rigorous FSC/SSC gating to exclude debris and cellular aggregates and select Cx3cr1‐GFPpos cells as compared to WT GFP negative control tissues. No DAPI or antibody labels were used in order to limit potential functional effects post transfer. (B) 5x10^4^ cells purified from 7 days PI cremasters, or saline, were injected into the anterior tibialis muscle of Cx3cr1‐GFPpos mice and left for 16 h before ischaemia (60 min) and reperfusion (120 min) was induced in the hind limb of the recipient mice by double ligation of the femoral artery. Tissue collection, weighing, enzymatic digestion and labelling of the cell suspension with DAPI, and fluorescent antibodies against CD45 and Ly6G (neutrophil specific clone 1A8) were used to analyse cell populations. [file PATH-240-291-s003.tif]
