# Supplementary material for: Underlying chronic inflammation alters the profile and mechanisms of acute neutrophil recruitment
Source: J Pathol. 2016 Oct 19;240(3):291–303. doi: 10.1002/path.4776 (PMC5082550; doi:10.1002/path.4776)
Supplement: Supplementary file 6 — Table S1. Intensity data for chemokine/cytokine immunoblot array of LPS‐stimulated cremasters. Chronic ischaemia, or sham surgery, was induced in the cremasters of WT mice. After 7 days, circulating neutrophils were depleted with anti‐Ly6G antibody (100 µg i.p. 24 h) and some tissues were monocyte/macrophage‐depleted with locally applied clodronate liposomes. Cremasters were subsequently stimulated with LPS (300ng i.s., 4 h) or saline. Tissues were collected, homogenised and analysed using a chemokine/cytokine array immunoblot according to manufacturer's instructions (R&D Systems). The mean intensity values for each chemokine/cytokine in each treatment group were normalised to total protein and the intensity of the control spots per blot. Each blot contained pooled tissue from two animals and was repeated twice. [file PATH-240-291-s004.doc]

**Table S1. Intensity data for chemokine/cytokine immunoblot array of LPS-stimulated cremasters.** Chronic ischaemia, or sham surgery, was induced in the cremasters of WT mice. After 7 days, circulating neutrophils were depleted with anti-Ly6G antibody (100 µg i.p. 24 h) and some tissues were monocyte/macrophage-depleted with locally applied clodronate liposomes. Cremasters were subsequently stimulated with LPS (300ng i.s., 4 h) or saline. Tissues were collected, homogenised and analysed using a chemokine/cytokine array immunoblot according to manufacturer’s instructions (R&D Systems). The mean intensity values for each chemokine/cytokine in each treatment group were normalised to total protein and the intensity of the control spots per blot. Each blot contained pooled tissue from two animals and was repeated twice.

|  | **Sham + saline** | **Sham + LPS** | **PI + saline** | **PI + LPS** | **PI + Clodronate + LPS** |
| --- | --- | --- | --- | --- | --- |
| Cxcl13 | 1698 | 1040 | 1655 | 1342 | 1473 |
| C5a | 7323 | 4703 | 9301 | 5902 | 8532 |
| G-CSF | 1013 | 6798 | 758 | 9170 | 7855 |
| GM-CSF | 639 | 1316 | 1035 | 1790 | 1786 |
| Ccl1/I-309 | 1416 | 1539 | 1042 | 2200 | 1761 |
| Ccl11/Eotaxin | 1514 | 859 | 745 | 1138 | 1354 |
| Icam-1 | 17865 | 13074 | 19085 | 13047 | 13903 |
| Ifn-γ | 1489 | 1368 | 1943 | 1659 | 2324 |
| Il-1a | 2511 | 10619 | 4141 | 14218 | 8305 |
| Il-1b | 1955 | 9252 | 2568 | 12387 | 10399 |
| Il-1ra | 10909 | 7148 | 19766 | 12513 | 9907 |
| Il-2 | 2823 | 1038 | 3215 | 1563 | 2401 |
| Il-3 | 1593 | 1397 | 780 | 1534 | 1157 |
| Il-4 | 1457 | 1232 | 1651 | 986 | 1267 |
| Il-5 | 792 | 1771 | 733 | 2223 | 1760 |
| Il-6 | 983 | 7236 | 644 | 12754 | 8525 |
| Il-7 | 1546 | 2762 | 1051 | 2862 | 2256 |
| Il-10 | 1453 | 1558 | 1077 | 2519 | 1472 |
| Il-12 p70 | 2003 | 2527 | 2849 | 2793 | 2764 |
| Il-13 | 1195 | 1445 | 1852 | 2359 | 2112 |
| Il-16 | 9835 | 8337 | 13834 | 13572 | 12182 |
| Il-17 | 1742 | 1894 | 2456 | 2890 | 3196 |
| Il-23 | 2222 | 1519 | 3917 | 2934 | 2289 |
| Il-27 | 2888 | 1016 | 3466 | 1393 | 1751 |
| Cxcl10/IP-10 | 1591 | 9571 | 1973 | 11016 | 9234 |
| Cxcl11/I-TAC | 1437 | 1811 | 1809 | 1875 | 1649 |
| Cxcl1/KC | 1122 | 13810 | 1223 | 14037 | 12373 |
| M-CSF | 3456 | 4480 | 4191 | 5397 | 5122 |
| Ccl2/JE/MCP-1 | 5837 | 25667 | 9107 | 20404 | 20047 |
| Ccl12/MCP-5 | 2892 | 6794 | 3388 | 9699 | 2713 |
| Cxcl9/MIG | 2965 | 12989 | 8196 | 14189 | 10211 |
| Ccl3/MIP-1α | 1942 | 11362 | 2832 | 16684 | 14035 |
| Ccl4/MIP-1β | 2187 | 9837 | 3164 | 16473 | 10072 |
| Cxcl2/MIP-2 | 2387 | 11067 | 2654 | 15385 | 15334 |
| Ccl5/RANTES | 2479 | 11171 | 5519 | 15036 | 8572 |
| Cxcl12/SDF-1 | 2580 | 548 | 2959 | 1025 | 1529 |
| Ccl17/TARC | 1259 | 1322 | 1361 | 1392 | 1194 |
| TIMP-1 | 13756 | 14299 | 17667 | 13722 | 12326 |
| Tnf | 1022 | 2371 | 1126 | 5636 | 3091 |
| TREM-1 | 1562 | 3373 | 2069 | 5573 | 8769 |
