# Supplementary material for: Underlying chronic inflammation alters the profile and mechanisms of acute neutrophil recruitment
Source: J Pathol. 2016 Oct 19;240(3):291–303. doi: 10.1002/path.4776 (PMC5082550; doi:10.1002/path.4776)
Supplement: Supplementary file 7 — Table S2. Intensity data for chemokine/cytokine immunoblot array from acute ischaemia/reperfusion‐stimulated hind limbs. Cx3cr1‐GFPpos cells were purified from 7 days PI cremasters by FACS. 5x10^4^ cells, or saline, were injected into the tibialis anterior muscle of Cx3cr1‐GFP or WT mice and left overnight. Acute ischaemia (60 min) and reperfusion (120 min) was induced buy double ligation of the femoral artery and vein. Muscles were collected, homogenised and analysed using a chemokine/cytokine array immunoblot according to manufacturer's instructions (R&D Systems). The intensity values for each chemokine/cytokine in each treatment group were normalised to total protein and the intensity of the control spots per blot. Each blot contained pooled tissues from 6 animals per group. [file PATH-240-291-s005.doc]

**Table S2. Intensity data for chemokine/cytokine immunoblot array from acute ischaemia/reperfusion-stimulated hind limbs.** Cx3cr1-GFP^pos^ cells were purified from 7 days PI cremasters by FACS. 5x10^4^ cells, or saline, were injected into the tibialis anterior muscle of Cx3cr1-GFP or WT mice and left overnight. Acute ischaemia (60 min) and reperfusion (120 min) was induced buy double ligation of the femoral artery and vein. Muscles were collected, homogenised and analysed using a chemokine/cytokine array immunoblot according to manufacturer’s instructions (R&D Systems). The intensity values for each chemokine/cytokine in each treatment group were normalised to total protein and the intensity of the control spots per blot. Each blot contained pooled tissues from 6 animals per group.

|  | Sham | **Sham + cell transfer** | **IR** | **IR + cell transfer** |
| --- | --- | --- | --- | --- |
| Cxcl13 | 4218 | 6877 | 6038 | 8163 |
| C5a | 10870 | 9104 | 9479 | 11781 |
| G-CSF | 3555 | 1932 | 1262 | 2008 |
| GM-CSF | 5694 | 2501 | 2281 | 3024 |
| Ccl1/I-309 | 7250 | 3468 | 3413 | 4358 |
| Ccl11/Eotaxin | 2568 | 1845 | 1975 | 2423 |
| Icam-1 | 17575 | 21557 | 17941 | 24940 |
| IFN-γ | 2687 | 1949 | 2019 | 2515 |
| Il-1a | 5984 | 5631 | 5257 | 6889 |
| Il-1b | 3312 | 2920 | 2317 | 3303 |
| Il-1ra | 11642 | 22470 | 15900 | 24157 |
| Il-2 | 4618 | 6078 | 4790 | 6856 |
| Il-3 | 2591 | 2133 | 3105 | 3340 |
| Il-4 | 3648 | 3442 | 3133 | 4158 |
| Il-5 | 3382 | 1455 | 1051 | 1579 |
| Il-6 | 3092 | 1822 | 1507 | 2102 |
| Il-7 | 4448 | 3316 | 4281 | 4835 |
| Il-10 | 4347 | 3042 | 3466 | 4132 |
| Il-12 p70 | 3926 | 6768 | 4753 | 7252 |
| Il-13 | 1222 | 2100 | 1937 | 2554 |
| Il-16 | 14051 | 11676 | 11724 | 14825 |
| Il-17 | 3860 | 2357 | 1960 | 2726 |
| Il-23 | 5374 | 6913 | 7134 | 8904 |
| Il-27 | 8281 | 8602 | 8008 | 10509 |
| Cxcl10/IP-10 | 1932 | 4796 | 5292 | 6402 |
| Cxcl11/I-TAC | 4075 | 4704 | 4515 | 5836 |
| Cxcl1/KC | 4088 | 3032 | 2264 | 3337 |
| M-CSF | 11423 | 8572 | 8510 | 10820 |
| Ccl2/JE/MCP-1 | 13479 | 15258 | 14948 | 19129 |
| Ccl12/MCP-5 | 5816 | 10044 | 8959 | 12014 |
| Cxcl9/MIG | 3759 | 7619 | 6530 | 8938 |
| Ccl3/MIP-1α | 2826 | 6979 | 4968 | 7523 |
| Ccl4/MIP-1β | 3901 | 2878 | 2186 | 3192 |
| Cxcl2/MIP-2 | 4955 | 5331 | 5403 | 6802 |
| Ccl5/RANTES | 4124 | 5982 | 7567 | 8620 |
| Cxcl12/SDF-1 | 15462 | 14042 | 16015 | 19087 |
| Ccl17/TARC | 1400 | 2595 | 3460 | 3856 |
| TIMP-1 | 16169 | 19006 | 15929 | 22061 |
| Tnf | 4770 | 3586 | 2430 | 3785 |
| TREM-1 | 5774 | 7987 | 7379 | 9721 |
